# The molecular interplay of the establishment of an infection – gene expression of *Diaphorina citri* gut and *Candidatus* Liberibacter asiaticus

**DOI:** 10.1186/s12864-021-07988-2

**Published:** 2021-09-21

**Authors:** Flavia Moura Manoel Bento, Josiane Cecília Darolt, Bruna Laís Merlin, Leandro Penã, Nelson Arno Wulff, Fernando Luis Cônsoli

**Affiliations:** 1 Insect Interactions Laboratory, Department of Entomology and Acarology, Luiz de Queiroz College of Agriculture, University of São Paulo, Avenida Pádua Dias 11, Piracicaba, São Paulo 13418-900 Brazil; 2 Fund for Citrus Protection (FUNDECITRUS), Araraquara, São Paulo 14807-040 Brazil; 3 Institute of Chemistry, São Paulo State University – UNESP, Araraquara, São Paulo Brazil; 4 Instituto de Biología Molecular y Celular de Plantas (IBMCP), Consejo Superior de Investigaciones Científicas (CSIC), Universidad Politécnica de Valencia (UPV), 46022 Valencia, Spain

**Keywords:** Host regulation, Effector proteins, Host – pathogen interactions, Microbial ecology, Infection strategies, Host immunity

## Abstract

**Background:**

*Candidatus* Liberibacter asiaticus (CLas) is one the causative agents of greening disease in citrus, an unccurable, devastating disease of citrus worldwide. CLas is vectored by *Diaphorina citri*, and the understanding of the molecular interplay between vector and pathogen will provide additional basis for the development and implementation of successful management strategies. We focused in the molecular interplay occurring in the gut of the vector, a major barrier for CLas invasion and colonization.

**Results:**

We investigated the differential expression of vector and CLas genes by analyzing a *de novo* reference metatranscriptome of the gut of adult psyllids fed of CLas-infected and healthy citrus plants for 1-2, 3-4 and 5-6 days. CLas regulates the immune response of the vector affecting the production of reactive species of oxygen and nitrogen, and the production of antimicrobial peptides. Moreover, CLas overexpressed *peroxiredoxin*, probably in a protective manner. The major transcript involved in immune expression was related to melanization, a *CLIP-domain serine protease* we believe participates in the wounding of epithelial cells damaged during infection, which is supported by the down-regulation of *pangolin*. We also detected that CLas modulates the gut peristalsis of psyllids through the down-regulation of *titin*, reducing the elimination of CLas with faeces. The up-regulation of the neuromodulator *arylalkylamine N-acetyltransferase* implies CLas also interferes with the double brain-gut communication circuitry of the vector. CLas colonizes the gut by expressing two *Type IVb pilin flp* genes and several chaperones that can also function as adhesins. We hypothesized biofilm formation occurs by the expression of the cold shock protein of CLas.

**Conclusions:**

The thorough detailed analysis of the transcritome of *Ca.* L. asiaticus and of *D. citri* at different time points of their interaction in the gut tissues of the host led to the identification of several host genes targeted for regulation by L. asiaticus, but also bacterial genes coding for potential effector proteins. The identified targets and effector proteins are potential targets for the development of new management strategies directed to interfere with the successful utilization of the psyllid vector by this pathogen.

**Supplementary Information:**

The online version contains supplementary material available at 10.1186/s12864-021-07988-2.

## Background

Bacterial plant pathogens are adapted to infect and propagate in insect tissues, as many insects are used as vectors by plant pathogens to infect new host plants and spread themselves in the environment. Plant pathogenic bacteria vectored by insects that use persistent, circulative, propagative mode of transmission require plastic phenotypes to interact with the different enviroments represented by sieves and tissues of the host plant, and the diverse environments faced in the gut lumen, hemocele and different tissues of the vector insect during the processes of acquisition and vector competence development [1]. The capability of these pathogens to infect completely different hosts (plant and insect), and yet to depend on the shuttle host (insect) to locate and collect the pathogen in a diseased plant, and later transport it to new, health host plants for the establishment of new infections requires a set of strategies devoted to manipulate the host insect [2, 3]. The understanding of the interactions pathogens and hosts have at their molecular level during the processes of host invasion and infection establishment is required for a fully comprehension of the mechanisms involved in guaranteeing the host – pathogen association. Such mechanisms of close interactions in the associations of host and pathogens represent potential new targets for the development of new technologies and/or use of existing technologies to interfere with the successful association pathogens establish with their hosts.

*Candidatus* Liberibacter are bacteria posing serious threat to food security worldwide by causing diseases to potatoes and citrus. Citrus is certainly the most severely damaged crop by *Ca.* Liberibacter infections, as three different species are known to infect citrus plants (*Ca.* L. asiaticus, *Ca.* L. americanus and *Ca.* L. africanus), causing the incurable greening or huanglongbing (HLB) disease [4–7]. *Candidatus* Liberibacter did not make to the top 10 plant pathogenic bacteria [8], but HLB has caused a tremendous impact in the citrus industry and led to the complete elimination of citrus orchards and the interruption of citrus production in what used to be highly productive citrus centers [7, 9, 10].

*Candidatus* Liberibacter asiaticus (CLas) is the most spread in the world, currently infecting citrus in the major producing areas [11–13]. In plants, *Candidatus* Liberibacter reside exclusively in the phloem sieve tubes [4, 14–16], but it will infect several tissues of their host vector insect [17–20]. CLas is vectored by the worldwidely distributed Asian Citrus Psyllid (ACP) *Diaphorina citri* Kuwayama (Hemiptera: Psyllidae) [4].

Despite the importance of this disease to the worldwide citriculture and all of the investments and efforts of the scientific community to understand the interactions of the pathogen with its vector, much of the mechanisms involved in the pathogen-vector interplay remain unknown. We learned that CLas is transmitted from host plant to host plant by establishing a persistent, propagative transmission mode with its vector, a transmission mode that is very common to plant viruses vectored by aphids and whiteflies [19, 21, 22]. CLas was also demonstrated to alter the host plant transcriptional profile [23–25]. We also learned CLas infects salivary glands and the midgut of vectors, tissues that are often recognized as natural barriers to circulative, propagative pathogens as they can prevent translocation of pathogens within the vector host [17, 22, 26, 27]. Differential gene expression analysis of CLas-infected psyllids also demonstrated CLas alters the gene expression profile of of the gut, salivary glands and whole-body of *D. citri* [19, 28–30]. More recently, new information on the higher efficiency of adults than psyllid nymphs as vectors of CLas has been reported, with adults displaying a higher successful rate of infection of healthy citrus plants than nymphs [27]. Nevertheless, little information at the physiological and molecular level on the interface of the interactions of CLas with key vector tissues is available [31].

The limitations in the availability of efficient, cost-effective strategies for the management of the vector and the disease prompted a large number of investigations for the exploitation of new technologies, and promising results were mainly obtained with RNAi-based approaches [32–35].

In here we focused on investigating the metatranscriptome of the gut of adults of *D. citri* feeding on CLas-infected citrus plants for different periods of time in order to identify genes of CLas that are expressed during the colonization of the gut of adults and the differential gene expression in the gut of psyllids over time against psyllids feeding on healthy, CLas-free citrus plants. Our major goal was to understand the dynamics of CLas gene expression during psyllid colonization and the response mechanisms that were activated in the gut epithelium of psyllids when exposed to *Ca*. Liberibacter asiaticus cells. We believe our data represents a source of very specific targets for the development and implementation of new strategies of psyllid/disease control using RNAi and/or gene editing technologies such as the CRISPR-Cas9 system.

## Results

### *De novo* transcriptome assembly

Sequencing from libraries of the gut of uninfected and CLas-infected nymphs and adults of *D. citri* yielded 395,151,161 reads, with an average of 16,464,632 reads/library. The use of the resulting 385,677,949 trimmed, quality-filtered reads (average 16,069,915 reads/library) allowed the *de novo* assembly of a transcriptome with 260,612,776 nucleotides (260 Mb), with transcripts with an average size of 481 bp and a N50 of 2,095 bp. The assembly resulted in 248,850 transcripts with an average of 39.9% GC content. Annotation of the transcriptome allowed the putative identification of 90,531 transcripts (36.4%), of which 52,081 transcripts were allocated to different gene ontology categories (Additional file 1). Additional filtering using the highest score hit after BlastX allowed the identification of 66,993 transcripts belonging to *D. citri*, 807 belonging to *Ca.* Liberibacter, and 1,967 to the secondary symbiont *Wolbachia*, among others (Additional file 2).

### CLas gene expression in the gut of adults of *Diaphorina citri*

Since we could not detect relevant read counts against transcripts belonging to *Ca*. L. asiaticus when using samples obtained from nymphs, only samples collected at the adult stage were subjected to differential expression analysis. Gene expression analysis recognized 807 transcripts belonging to *Ca*. L. asiaticus in the gut of adult psyllids fed on CLas-infected citrus plants in at least one of feeding exposure periods analyzed for adults (1-2 d; 3-4 d; 5-6 d - *A1CLas*^*+*^, *A2CLas*^*+*^ and *A3CLas*^*+*^, respectively), with no CLas-related transcripts being identified in gut samples from insects fed on healthy plants (*CLas*^*-*^).

**Fig. 1:**
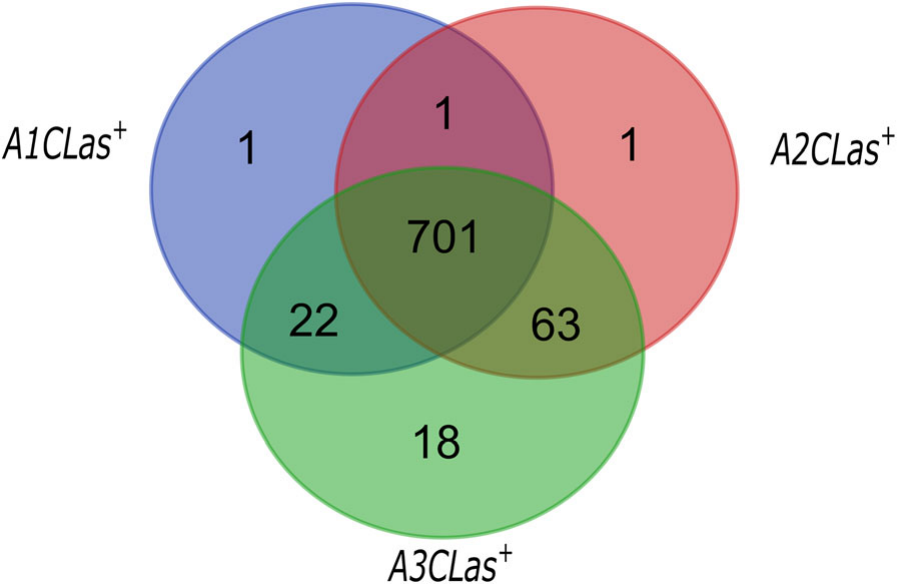
Venn diagram of *Candidatus* Liberibacter asiaticus transcripts differentially expressed in gut of adults at different periods of feeding on CLas-infected citrus plant. *A1CLas*^*+*^: adults that fed on CLas-infected citrus plant for 1-2 days; *A2CLas*^*+*^: adults that fed on CLas-infected citrus plant for 3-4 days; and *A3CLas*^*+*^: adults that fed on CLas-infected citrus plant for 5-6 days.

The detection of gene expression of *Ca.* L. asiaticus in adult psyllids increased in adults feeding on CLas-infected plants for 1-2 d to 5-6 d. Thus, 725 transcripts of CLas were expressed in *A1CLas*^*+*^, 766 in *A2CLas*^*+*^ and 804 in *A3CLas*^*+*^ (Fig. 1). One transcript was expressed only in *A1CLas*^*+*^ and *A2CLas*^*+*^, 22 in *A1CLas*^*+*^ and *A3CLas*^*+*^, and 63 in *A2CLas*^*+*^ and *A3CLas*^*+*^. We also identified CLas genes that were exclusively expressed at the early (1-2 d, *A1CLas*^*+*^= 1 transcript, DN14421_c0_g2_i2 = nicotinate-nucleotide adenylyltransferase), intermediate (3-4 d, *A2CLas*^*+*^= 1 transcript, DN18703_c1_g1_i24 = Flp type IVb pilin) and late stage (5-6 d, *A3CLas*^*+*^= 18 transcripts) after adult started feeding on CLas-infected citrus plants (Fig. 1). Most of these transcripts were represented by several isoforms of a gene, with different isoforms expressed in more than one of the sampled feeding times. Only *A3CLas*^*+*^ adults had transcripts represented by unique isoforms of a gene specifically expressed in their gut (Table 1).

**Table 1:** Single isoforms of CLas transcripts exclusively expressed in the gut of adult psyllids in *A3CLas+*

Transcript ID	Annotation	TPM count
DN1443_c0_g1_i1	hypothetical protein PSGCA5_05	58.77
DN15414_c0_g1_i2	pyridoxine 5'-phosphate synthase	5.33
DN25987_c0_g1_i1	hypothetical protein	30.77
DN32802_c0_g1_i1	anti-repressor protein	38.31
DN39169_c0_g1_i1	hypothetical protein BWK56_05555	69.16
DN44737_c0_g1_i1	threonine--tRNA ligase	168.28
DN47430_c0_g1_i1	flagellar basal body rod protein FlgF	54.15
DN55271_c0_g1_i1	phage related protein	10.66
DN62541_c0_g1_i1	flagellar basal body P-ring protein FlgI	31.40
DN64078_c0_g1_i1	putative phage-related acetyltransferase	43.56

Pairwise gene expression analysis of CLas in the gut of adult psyllids after different periods of feeding on CLas-infected citrus plants revealed a high number of differentially expressed CLas genes in *A3CLas*^*+*^ as compared to *A1CLas*^*+*^ and *A2CLas*^*+*^ adults (Additional file 3). One-hundred transcripts out of the over 700 transcripts detected in the three sampling times differed in their abundance (Additional file 3). Differences in the level of expression were detected for 80 transcripts when comparing *A1CLas*^*+*^ and *A3CLas*^*+*^ and 20 transcripts in comparisons of *A2CLas*^*+*^ and *A3CLas*^*+*^ (Additional file 3). CLas expression was always higher in *A3CLas*^*+*^ when compared to the others. No differences in gene expression between *A1CLas*^*+*^ and *A2CLas*^*+*^ were detected (Additional file 3).

### Differential gene expression in the gut of *CLas*^*+*^ and *CLas*^*-*^ adults of *Diaphorina citri*

Differential gene expression (DE) of the gut of adults of *D. citri* after feeding on CLas-infected plants for 1-2 days identified 24 DE transcripts in the gut of *CLas*^*+*^ insects, 20 of them were up-regulated and 4 were down-regulated in response to CLas infection. Most of the up-regulated transcripts (11) belong to uncharacterized proteins, while nine up-regulated transcripts were identified (Table 2). After 3-4 days of feeding on CLas-infected plants, the number of DE transcripts in the gut of *CLas*^*+*^ as compared to *CLas*^*-*^ adult psyllids increased to 35, most of which were up-regulated (27). Eighteen out of the 27 up-regulated transcripts, and seven out of the eight down-regulated transcripts were putatively identified (Table 2). The number of DE transcripts in the gut of adult psyllids after 5-6 days of feeding on CLas-infected plants was the highest, with 61 DE transcripts in the gut of *CLas*^*+*^ insects, 57 (33 unknown proteins) of them were up-regulated and 4 were down-regulated when compared to *CLas*^*-*^ adult psyllids (Table 2).

**Table 2:** Comparative differential gene expression of adult psyllids fed on CLas infected and non-infected citrus plants for different periods of time (A1= 1-2 d; A2= 3-4 d; A3= 5-6 d)

Stage	DE	Annotation	Transcript ID	FC	FDR
*A1 - *CLas*^*+*^ x *CLas*^*-*^	Up-regulated	arylsulfatase B-like isoform X1	DN19237_c6_g3_i2	20.9	2.0*e*^*-*2^
		CLIP domain-containing serine protease 2-like isoform X1	DN16851_c1_g2_i2	119.9	3.0*e*^-3^
		kinesin-like protein KIF3B isoform X4	DN10161_c0_g1_i1	55.2	6.1*e*^-3^
		membrane metallo-endopeptidase-like 1 isoform X2	DN16798_c0_g1_i1	49.0	4.8*e*^-4^
		methylglutaconyl-CoA hydratase, mitochondrial	DN18628_c4_g1_i2	26.7	1.0*e*^-2^
		mitochondrial cytochrome c oxidase subunit IV	DN18774_c0_g1_i7	44.2	2.3*e*^-6^
		nuclear speckle splicing regulatory protein 1-like	DN17904_c2_g1_i3	133.9	4.0*e*^-2^
		uncharacterized protein LOC103515486	DN16253_c6_g2_i4	11.3	9.64*e*^-3^
		protein phosphatase 1 regulatory subunit 21	DN19112_c0_g1_i1	36.7	0.02
		putative rna-directed dna polymerase from transposon bs	DN20114_c0_g1_i11	26.6	0.05
		uncharacterized protein LOC103508016	DN17144_c6_g5_i2	83.6	2.93*e*^-5^
		uncharacterized protein LOC103508016	DN17144_c6_g5_i3	62.7	0.02
		uncharacterized protein LOC103518490	DN15191_c0_g1_i1	222.8	2.03*e*^-3^
		uncharacterized protein LOC103518490	DN15191_c0_g1_i4	41.0	6.75*e*^-3^
		uncharacterized protein LOC103519237	DN15191_c0_g1_i7	210.2	1.06*e*^-4^
		uncharacterized protein LOC113469268	DN20635_c0_g3_i1	242.2	1.06*e*^-4^
		uncharacterized protein LOC113469268	DN20635_c0_g1_i3	106.3	5.87*e*^-4^
		uncharacterized protein LOC113471433	DN20585_c3_g1_i12	167.6	5.28*e*^-3^
		uncharacterized protein PFB0145c-like	DN15546_c0_g1_i1	31.3	0.04
		uncharacterized protein YMR317W-like	DN15237_c0_g1_i1	132.6	4.69*e*^-4^
	Down-regulated	NADH dehydrogenase [ubiquinone] 1 beta subcomplex subunit 3	DN17549_c4_g1_i2	-32.5	6.75*e*^-3^
		protein pangolin, isoforms A/H/I/S-like	DN15424_c0_g3_i2	-28.0	0.03
		titin isoform X7	DN21409_c5_g2_i1	-36.1	0.03
		zonadhesin-like	DN14644_c0_g1_i1	-13.0	0.03
**A2 -*CLas*^*+*^ x *CLas*^*-*^	Up-regulated	Ac1147-like protein	DN18394_c1_g1_i3	19.7	0.02
		arylalkylamine N-acetyltransferase	DN20634_c1_g1_i13	45.9	0.05
		cathepsin L1-like	DN15698_c4_g11_i2	111.4	1.45*e*^-3^
		hypothetical protein DAPPUDRAFT_37179, partial	DN18534_c0_g5_i1	17.0	7.51*e*^-4^
		hypothetical protein g.47064	DN18473_c2_g1_i1	11.7	8.68*e*^-3^
		hypothetical protein g.7198	DN16128_c4_g1_i1	11.1	3.86*e*^-3^
		large neutral amino acids transporter small subunit 1	DN20157_c2_g3_i2	66.6	1.50*e*^-4^
		nocturnin isoform X1	DN15927_c10_g1_i6	15.1	2.82*e*^-4^
		predicted protein	DN21032_c17_g1_i2	27.0	2.19*e*^-3^
		predicted protein	DN21032_c17_g1_i1	23.9	6.41*e*^-3^
		uncharacterized protein LOC105736982	DN21288_c9_g7_i2	28.5	8.64*e*^-4^
		uncharacterized protein LOC103515486	DN16253_c6_g2_i4	12.6	0.04
		profilin	DN19422_c2_g2_i1	162.4	7.00*e*^-9^
		protein hu-li tai shao	DN19744_c0_g2_i2	12.2	0.01
		putative senescence-associated protein	DN18133_c2_g1_i3	28.0	1.41*e*^-3^
		Regulator of rDNA transcription protein 15	DN16244_c2_g3_i1	24.8	1.62*e*^-3^
		Regulator of rDNA transcription protein 15	DN21288_c9_g7_i1	16.4	6.43*e*^-3^
		Regulator of rDNA transcription protein 15	DN16244_c2_g3_i2	15.6	7.58*e*^-4^
		Regulator of rDNA transcription protein 15	DN16244_c2_g3_i3	13.0	5.46*e*^-3^
		reverse transcriptase-like protein	DN21162_c2_g1_i4	12.4	0.02
		sodium/potassium-transporting ATPase subunit alpha-like	DN14485_c0_g1_i1	17.9	5.33*e*^-4^
		UNC93-like protein MFSD11	DN16539_c2_g6_i1	48.5	0.04
		Uncharacterized protein ART2	DN19721_c0_g1_i3	9.8	0.02
		uncharacterized protein LOC105206152	DN18133_c2_g1_i1	22.8	1.45*e*^-3^
		uncharacterized protein LOC105693632	DN19721_c0_g4_i1	7.9	8.56*e*^-3^
		uncharacterized protein LOC113219380	DN19326_c1_g1_i2	35.7	6.21*e*^-4^
		uncharacterized protein LOC113469268	DN20635_c0_g1_i1	37.4	0.02
	Down-regulated	leptin receptor overlapping transcript-like 1	DN20058_c2_g3_i4	-26.7	0.02
		methylglutaconyl-CoA hydratase, mitochondrial	DN18628_c4_g1_i2	-11.3	1.27*e*^-3^
		putative juvenile hormone binding protein	DN21423_c1_g12_i2	-12.3	0.04
		Soluble NSF attachment protein	DN21196_c2_g1_i1	-19.8	5.64*e*^-7^
		translation elongation factor 2	DN21188_c1_g7_i2	-11.9	0.02
		tubulin-folding cofactor B	DN17504_c3_g2_i6	-4.5	1.82*e*^-3^
		uncharacterized protein LOC103513992	DN16335_c5_g1_i1	-16.7	5.18*e*^-3^
		zinc transporter ZIP10 isoform X1	DN19132_c8_g1_i5	-43.4	0.02
***A3 - *CLas*^*+*^ x *CLas*^*-*^	Up-regulated	2-oxoglutarate dehydrogenase, mitochondrial-like	DN14833_c0_g1_i1	8.6	0.03
		Ac1147-like protein	DN21090_c8_g2_i1	50.3	0.04
		BCL2/adenovirus E1B 19 kDa protein-interacting protein 3	DN20168_c2_g3_i4	21.0	0.04
		beta-1,3-galactosyltransferase 5-like	DN18243_c2_g2_i2	20.2	7.31*e*^-3^
		cleavage and polyadenylation specificity factor subunit CG7185-like	DN15201_c0_g1_i1	15.6	6.05*e*^-3^
		cytochrome P450-like TBP	DN19721_c0_g1_i4	35.7	0.05
		dynein heavy chain 7, axonemal-like	DN35915_c0_g1_i1	17.3	0.05
		gamma-glutamyl hydrolase-like	DN18300_c5_g1_i2	16.7	3.96*e*^-5^
		GATA zinc finger domain-containing protein 14-like	DN16387_c3_g2_i1	12.2	6.66*e*^-3^
		glycerophosphocholine phosphodiesterase GPCPD1-like isoform X1	DN15741_c0_g2_i2	41.6	1.10*e*^-4^
		hydrocephalus-inducing protein homolog	DN14616_c0_g1_i2	26.6	0.03
		hydrocephalus-inducing protein homolog	DN14616_c0_g2_i2	16.6	0.02
		hypothetical protein ALC57_18598	DN17391_c0_g1_i2	230.6	1.56*e*^-4^
		hypothetical protein FF38_03795	DN18516_c0_g3_i1	67.0	0.02
		hypothetical protein LOTGIDRAFT_202939	DN17968_c0_g2_i3	101.2	0.03
		hypothetical protein Phum_PHUM590900	DN17430_c2_g2_i1	24.2	0.02
		keratin-associated protein 5-3-like	DN15230_c0_g1_i2	14.2	6.76*e*^-4^
		keratinocyte proline-rich protein-like	DN20284_c2_g1_i3	11.5	0.02
		mucin-5AC-like, partial	DN16207_c6_g5_i1	7.7	0.04
		uncharacterized protein LOC103511862	DN17183_c5_g2_i3	9.4	0.04
		uncharacterized protein LOC103513455	DN17270_c3_g2_i1	46.8	0.04
		uncharacterized protein LOC103514633	DN18467_c1_g5_i1	31.7	3.61*e*^-5^
		uncharacterized protein LOC103515486	DN16253_c6_g2_i4	12.6	0.04
		uncharacterized protein LOC103518002	DN18156_c6_g2_i2	10.0	0.03
		uncharacterized protein LOC103521603	DN17270_c3_g2_i2	63.7	0.01
		uncharacterized protein LOC103521674	DN15182_c0_g1_i1	9.3	0.03
		protein AMN1 homolog	DN15340_c0_g1_i1	8.9	0.04
		protein FAM133-like isoform X1	DN19474_c1_g3_i11	15.4	8.40*e*^-3^
		protein PRRC2C-like	DN15390_c0_g1_i1	21.0	3.47*e*^-4^
		protein suppressor of forked	DN20501_c1_g1_i2	50.7	0.04
		putative juvenile hormone binding protein	DN21423_c1_g12_i1	33.0	5.4*e*^-5^
		putative juvenile hormone binding protein	DN21423_c1_g12_i2	9.4	8.34*e*^-3^
		RNA-directed DNA polymerase from mobile element jockey-like	DN19725_c3_g1_i3	14.1	6.95*e*^-3^
		senescence-associated protein	DN16041_c1_g1_i2	198.7	1.67*e*^-4^
		SWI/SNF chromatin-remodeling complex subunit SNF5-like	DN18520_c6_g1_i1	52.5	1.18*e*^-5^
		uncharacterized protein Dyak_GE27401	DN16348_c2_g1_i1	18.0	0.02
		uncharacterized protein LOC103507494 isoform X2	DN20548_c5_g2_i7	13.1	0.01
		uncharacterized protein LOC103507496	DN20260_c2_g2_i2	17.0	6.6*e*^-4^
		uncharacterized protein LOC103507787	DN20585_c3_g1_i2	60.0	8.58*e*^-3^
		uncharacterized protein LOC103512709	DN20458_c5_g2_i2	29.7	0.03
		uncharacterized protein LOC103514651	DN18692_c1_g1_i7	41.1	9.13*e*^-4^
		uncharacterized protein LOC103516995	DN14754_c0_g3_i1	39.3	2.89*e*^-3^
		uncharacterized protein LOC103518490	DN15191_c0_g1_i2	105.3	0.05
		uncharacterized protein LOC103518490	DN15191_c0_g1_i4	85.7	7.47*e*^-3^
		uncharacterized protein LOC103519237	DN15191_c0_g1_i7	36.9	0.01
		uncharacterized protein LOC103520317	DN17514_c8_g2_i1	10.7	0.01
		uncharacterized protein LOC103524424	DN14888_c0_g2_i1	7.8	0.04
		uncharacterized protein LOC108252545 isoform X1	DN16387_c3_g1_i9	7.6	0.03
		uncharacterized protein LOC113219351	DN17193_c2_g1_i6	74.5	8.74*e*^-4^
		uncharacterized protein LOC113219380	DN18715_c0_g1_i6	305.6	9.53*e*^-5^
		uncharacterized protein LOC113219380	DN21090_c7_g10_i1	82.0	0.04
		uncharacterized protein LOC113469268	DN20635_c0_g3_i2	73.8	0.03
		uncharacterized protein LOC113469268	DN20635_c0_g1_i1	37.4	0.02
		uncharacterized protein LOC113469268	DN20635_c0_g3_i1	36.9	0.02
		uncharacterized protein LOC113469388	DN14497_c0_g1_i2	8.9	0.04
		uncharacterized protein YMR317W-like	DN15237_c0_g1_i1	60.2	8.52*e*^-4^
		Zgc:165536 protein	DN19721_c0_g2_i1	186.9	8.52*e*^-4^
	Down-regulated	annexin B9-like isoform X1	DN19784_c8_g1_i2	-11.0	0.03
		cytosolic purine 5'-nucleotidase isoform X4	DN16811_c2_g1_i5	-37.4	0.02
		probable 4-coumarate--CoA ligase 1	DN19422_c2_g3_i12	-7.4	0.05
		UDP-glucuronosyltransferase 2C1-like	DN20095_c3_g1_i5	-12.4	1.92*e*^-3^

* A1- *CLas+* x *CLas-*: Differential gene expression of adults with 1 to 2 days of feeding; ** A2- *CLas+* x *CLas-*: Differential gene expression of adults with 3 to 4 days of feeding; *** A3- *CLas+* x *CLas-*: Differential gene expression of adults with 5 to 6 days of feeding

**Fig. 2:**
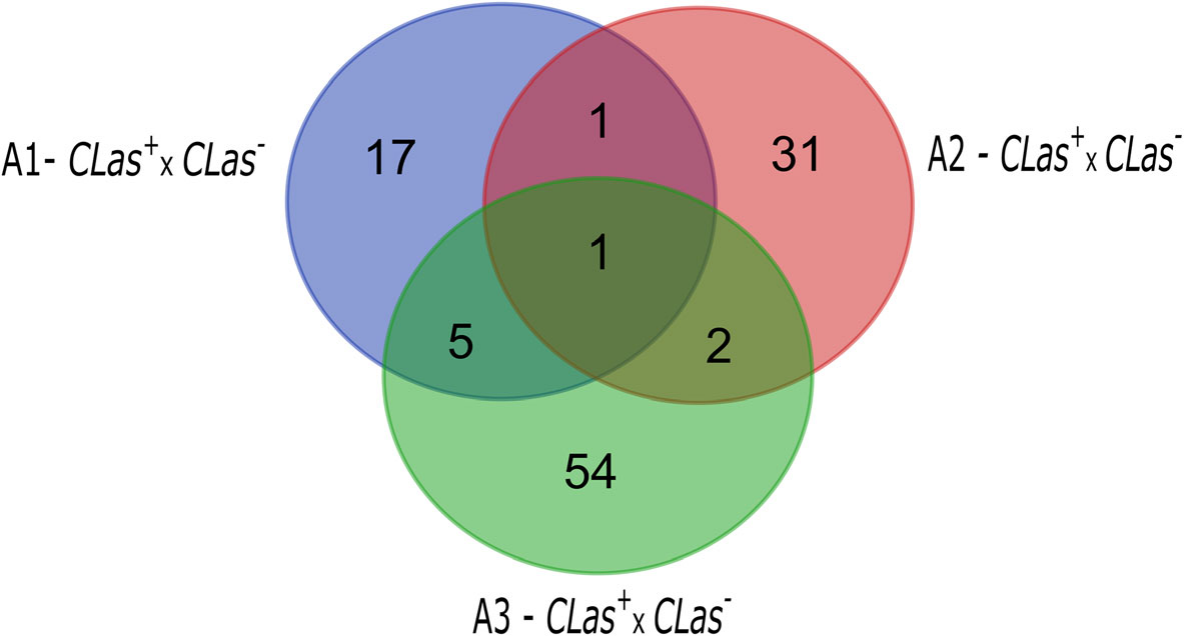
Venn diagram of *Diaphorina citri* transcripts differentially expressed in gut of adults at different periods of feeding on CLas-infected citrus plant compared to insects that fed on healthy citrus plant. A1 - *CLas*^*+*^ x *CLas*^*-*^: adults that fed on CLas-infected versus uninfected citrus plant for 1-2 days; A2 - *CLas*^*+*^x *CLas*^*-*^: adults that fed on CLas-infected versus uninfected for 3-4 days; and A3 *- CLas*^*+*^x *CLas*^*-*^: adults that fed on CLas-infected versus uninfected for 5-6 days.

From the total number of differentially expressed transcripts of *CLas*^*+*^ when compared to their respective *CLas*^*-*^ psyllids, only one transcript (DN16253_c6_g2_i4: uncharacterized protein LOC103515486) was differentially expressed in *CLas*^*+*^ psyllids regardless the time they were allowed to feed on CLas-infected citrus plants as compared to psyllids fed on CLas-uninfected citrus plants (Fig. 2). Most of the DE transcripts detected in *CLas*^*+*^ psyllids differed only at the specific stage they were compared. Seventeen (71%) of the DE transcripts detected in the gut of adult psyllids after 1-2 d, 31 (88%) after 3-4 d and 60 (88%) after 5-6 d of feeding in CLas-infected plants differed from controls specifically at each particular stage (Fig. 2).

## Discussion

The differential gene expression of the gut of adult psyllids differed depending on the duration of feeding on CLas-infected citrus plants. The number of DE transcripts in the gut of psyllids increased as the time the psyllids fed on CLas-infected plants increased. Most interesting, the majority of DE transcripts detected were specific to each one of the periods of feeding psyllids remained exposed to CLas-infected plants.

Gene expression of CLas in the gut of adult psyllids after different times of feeding on CLas-infected citrus plants were quite different. CLas transcription in the gut of adult psyllids was highly active soon after adult feeding started, but expression of a large set of genes was significantly increased at later stages of feeding (*A3CLas*^*+*^).

### Immune attack and immune defense responses

Bacteria that gain access to the hemocoel of insects find an easy way through the gut epithelium of the midgut, since the epithelium in this region does not have the cuticle lining protecting the fore- and the hindgut. Psyllids, as other sap-feeding insects do not carry the peritrophic membrane that protects the midgut epithelium of several other groups of insects [36, 37]. But regardless the presence of such physical barriers, the front line of defense of the gut immune system involves the activation of the epithelial innate immune system for the production of reactive oxygen species (ROS). Bacteria that survive in the gut of insects are either resistant to or are able to metabolize ROS [38, 39].

Analysis of the CLas gene expression in the gut of adult psyllids demonstrated that CLas expressed the cytoprotective antioxidant enzyme peroxiredoxin (DN17546_c4_g1_i1), capable of reducing ROS and reactive nitrogen species (RNS) produced in the process of gut infection [40, 41]. Peroxiredoxins (EC 1.11.1.15) are cysteine-dependent peroxidases targeting hydrogen peroxide, peroxynitrite and organic peroxide as substrates [42]. Peroxiredoxins utilize either 2-Cys or 1-Cys reaction catalysis mechanisms [42], with CLas showing two chromosomal 1-Cys-1 peroxiredoxin genes (CLIBASIA_RS00940 and CLIBASIA_RS00445). These genes are highly conserved in all CLas sequenced strains [42]. The DE expressed transcript detected in the gut of CLas-infected psyllids (DN17546_c4_g1_i1) shared 100% identity with CLIBASIA_RS00940 (WP_012778530). But peroxidase activity has only been reported for CLIBASIA_RS00445 (WP_012778432.1) [42, 43]. The CLas BCP endoded by this gene was demonstrated to be an extracellular 1-Cys peroxiredoxin that suppresses the signaling and accumulation of oxypilins in plants mediated by the respiratory burst oxidase homologs (RBOH). Regulation of this process was reported as essential for CLas survival and host plant colonization [42, 43].

The significant increase in the expression of peroxiredoxin from *A1CLas*^*+*^ to *A3CLas*^*+*^ (FC = 13.48) suggests this enzyme provides an increased contribution in the infection process, as CLas cells also established an intracellular interaction with the gut epithelium of psyllids. CLas expression of peroxiredoxin occurred despite the expected reduction of ROS in the gut epithelium of adult psyllids, as the major source for ROS production in the gut, NADH dehydrogenase [ubiquinone] 1 beta subcomplex subunit 3 (DN17549_c4_g1_i2), was down-regulated in *CLas*^*+*^ psyllids.

These results suggest that CLas can inhibit hydrogen peroxide production in the host insect, and that peroxiredoxin is certainly serving roles other than its antioxidative contribution. The adipokinetic hormone (ADK) is involved in the regulation of the oxidative stress response in insects [44]. Unfortunately, the expression levels of ADK-related genes by CLas infection could not be verified as ADK is produced and stored in neurosecretory cells of the *corpora cardiaca*. Nevertheless, regulation of this neuropeptide hormone in the gut epithelium of *CLas*^*+*^ psyllids could occur by the up-regulation of the metallo-endopeptidase-like 1 isoform X2 (DN16798_c0_g1_i1) neprilysin. Neprilysins are metalloproteases better known for their neuropeptide degrading activity, while also carrying peptide-degrading activity in other tissues, including the gut [45].

CLas peroxiredoxin could also be acting in the regulation of H_2_O_2_-mediated cell signaling processes, such as those involved in growth and immune responses [46]. The oxidative response produced by gut epithelia due to microbial infection leads to cell proliferation and modulation of the innate immune response [47], and ROS is required for inducing nitric oxide production in the gut. Nitric oxide production will in turn trigger the synthesis of antimicrobial peptides (AMP) and activate organ-to-organ communication [48]. Therefore, the down-regulation of ROS production in the gut of *CLas*^*+*^ psyllids could explain the lack of differential expression of genes belonging to the AMP production pathways. AMP synthesis can also be elicited by the recognition of peptidoglycans released by bacteria by membrane-bound peptidoglycan recognition proteins if peptidoglycans survive to amidase degradation [39].

There is very little information on insect gut phagocytosis, although proteomic analysis of anal droplets of the beetle *Cryptorhynchus lapathi* larvae led to the identification of several proteins to support an immune cellular response at the gut level [49]. Moreover, phagocytic receptors identified in the gut epithelium of *Drosophila* were proven to play crucial role in the phagocytosis of both Gram bacteria, and in controlling hemocele infection by bacteria invasion through the gut epithelium [50]. We could not detect any changes at the molecular level in *CLas*^*+*^ psyllids that would demonstrate a phagocytic response in the gut, as well as we did not observe adult psyllids to build an immune response to CLas infection by activating ROS and AMP pathways.

Nevertheless, differential gene expression analysis of the gut of *A1CLas*^*+*^ psyllids demonstrated the mounting of a defensive response of the gut epithelium based on the increased expression of a CLIP domain-containing serine protease 2-like (DN16851_c1_g2_i2). CLIP domain-containing serine protease 2-like has been recently reported to be up-regulated in the midgut of CLas-infected psyllids [29]. Serine proteases containing a CLIP domain are involved in the regulation of humoral responses through the activation of prophenoloxidases (PPO) and the Toll immune signaling pathway. Activation of Toll pathway leads to antimicrobial peptides production, while the activation of PPO results in melanogenesis [51, 52]. Toll activation requires a multi-step proteolytic cascade [53], and the lack of additional differentially expressed serine proteases and the antimicrobial peptides transcripts in the psyllid transcriptome support the argument that the up-regulation of CLIP-domain serine protease in the gut of ACP feeding on CLas-infected plants leads to the activation of melanization as an immune response against CLas infection. We also believe the activation of melanization at this stage of psyllid-CLas interaction was triggered by a local response caused by cell injury and invasion of the gut epithelium by CLas cells, resulting in the production of wound clots in order to avoid infected, damaged cells to suffer further damage, die and be replaced by *nidi* cells. This hypothesis is also supported by the down-regulation of pangolin (*Pan*) (DN15424_c0_g3_i2), a key regulator of the Wnt/Wg pathway. Wnt proteins are highly conserved and participate in the control of growth, patterning, tissue and energy homeostasis. Pangolin was reported to bind to the β-catenin homologue Armadillo, acting directly in the regulation of gene expression in response to Wnt signaling [54, 55]. The Wnt signaling in *Drosophila* is an important process for the homeostasis of the gut tissue as it is involved in the regeneration of adult gut epithelial cells [56, 57]. Thus, the down-regulation of pangolin interferes with the activation of the Wnt signaling to regulate gene expression involved in the replacement of CLas-infected cells by the activation of the nidi cells to differentiate into new, active cells of the gut epithelium.

Studies on the role of genotype-genotype interactions of insects and parasites demonstrated activation and differential gene expression of the host immune machinery depending on the interacting parasite genotype. Alternative splicing was also reported as a required element in the specificity of the immune response of insects to specific parasite genotypes [58]. The nuclear speckles carry pre-mRNA splicing machinery composed by nuclear speckle-related proteins that mediate alternative splice site selection in targeted pre-mRNAs [59, 60]. The detected up-regulation of proteins that mediate alternative splice site selection in targeted mRNAs, the nuclear speckle splicing regulatory protein 1-like (DN17904_c2_g1_i3) in *CLas*^*+*^ psyllids, suggests CLas acts on the regulation of gene expression of infected psyllids at the molecular level.

The psyllid initial defense response against CLas infection also included the up-regulation of arylsulfatase B (DN19237_c6_g3_i2), an enzyme stored in lysosomes that acts on large glycosaminoglycan molecules by removing attached sulfate groups [61]. Glycosaminoglycans are components of proteoglycans commonly exploited by pathogens as receptors for their adherence to different tissues [62]. Glycosaminoglycans are produced by some pathogenic bacteria as extracellular capsule and used in the process of host infection and colonization to facilitate pathogen attachment, invasion and/or evasion of host defensive mechanisms [63]. Arylsulfatases B can also participate in processes of biotransformation, mediating the sulfonation of xenobiotics by other enzymes to facilitate their excretion [64]. In this case, increase in the arylsulfatase could be related to the psyllid physiological needs to metabolize the high levels of flavonoids (hesperidin, narirutin and dydimin) produced in citrus plants infected by *Ca.* Liberibacter asiaticus [65–67]. Flavanoids are important metabolites produced by plants in response to abiotic and biotic stressors and play a relevant contribution in plant resistance against microbes and herbivores [68], including citrus plants in response to CLas infection [69, 70].

The overexpression of immune related genes and the required increased cell metabolism early in the process of interaction of the psyllid gut epithelium with CLas can explain the up-regulation of the mitochondrial cytochrome c oxidase subunit IV (DN18774_c0_g1_i7) in *CLas*^*+*^ insects. Mitochondrial cytochrome c oxidase subunit IV is the major regulation site for oxidative phosphorylation by catalyzing the final step of electron transfer in mitochondria [71].

### Cross-talk in the colonization of the gut lumen

Bacteria that survive the harsh chemical environment and the immune barriers available in the gut will have to use strategies to avoid their rapid elimination from the gut with faeces [39, 72]. Insects are active feeders and food fastly transits through the gut. The fast transit of the food in the gut has been argued as one condition to explain the controversial lack of a resident microbiome in lepidopteran larvae, for example [73].

CLas seems to employ different strategies to colonize the gut lumen by avoiding its elimination with faeces: regulation of gut peristalsis, synthesis of adherence proteins, and biofilm formation.

CLas regulation of the psyllid gut peristalsis is thought to occur through the up-regulation of the host neprilysin as earlier discussed. Neprilysins are reported to degrade peptide hormones, and the gut of insects contains several hormone-producing cells. Peptide hormones produced in the gut act as signaling molecules that are involved in the regulation of a range of processes, including gut peristalsis [74–76]. The hypothesis that CLas modulates the gut peristalsis of psyllids is supported by the down-regulation of titin (DN21409_c5_g2_i1) transcription in *CLas*^*+*^ psyllids. Muscle degeneration is marked by a reduction in titin proteins, and gut-associated muscles are in charge of producing the peristaltic movement observed in the gut. Additionaly, the intracellular non-catalytic domain of neprilysins in excess is shown to induce muscle degeneration [77]. Thus, the degeneration of muscles associated with the gut would certainly impair gut peristalsis and consequently reduce the elimination of bacteria with faeces [39, 72].

Regulation of signaling in the gut is also evidenced by the up-regulation of arylalkylamine N-acetyltransferase (DN20634_c1_g1_i13) transcription in *A2CLas*^*+*^ psyllids. Arylalkylamine N-acetyltransferases are involved in the N-acetylation of arylalkylamines, playing an important role in the synthesis of melatonin in vertebrates and invertebrates [78, 79]. But in insects, arylalkylamine N-acetyltransferases also inactivate arylalkymines that play important roles as neuromodulators, such as octopamine, dopamine and serotonin [80, 81]. These neuromodulators are also implicated in the double brain-gut communication circuitry [82, 83].

CLas adherence to the psyllid gut was achieved by the expression of three genes of a subtype of the type IVb pilins, the tight adherence pili (Tad = Flp). Flp pilins are common to several Gram^+^ and Gram^-^ bacteria. Type IV pili are highly diverse and involved in a number of protein-protein interactions, but flp pili are better known for their role in adherence to living and nonliving surfaces. Pili are also involved in cell motility, secretion of exoproteins, and in host cell manipulation under extreme conditions [84, 85].

The expression of Type IVc pili of *Ca.* L. asiaticus was reported to be higher in psyllids than in plants, but only one (*flp3 pilin*) out of five *flp* genes was reported to be up-regulated in psyllids [86]. The *flp3 pilin* gene was demonstrated to be under the regulation of VisN and VisR, demonstrating these proteins are important in the colonization of the host vector [86]. In our analysis of the transcriptional profile of CLas in the gut of adult psyllids, we detected the expression of several isoforms of two *flp* genes (DN14425_c0_g1; DN18703_c0_g1), but both belonging to the family Type IVb pilin. Three isoforms of gene DN14425_c0_g1 (DN14425_c0_g1_i2; DN14425_c0_g1_i3; DN14425_c0_g1_i4) and two of gene DN18703_c0_g1 (DN18703_c0_g1_i21 and DN18703_c0_g1_i23) were highly and consistently expressed in the gut of psyllids in all sampling periods. Three CLas LuxR family transcriptional regulators (DN1177_c0_g1_i1, DN14940_c0_g2_i1, and DN38633_c0_g1_i1) were also observed in the gut of *CLas*^*+*^ psyllids; one of them refers to the VisR regulator (DN1177_c0_g1_i1 = CLIBASIA_02900), and the other two to the VisN regulator (DN14940_c0_g2_i1 and DN38633_c0_g1_i1 = CLIBASIA_02905) of flp3 pilin gene reported by Andrade & Wang [86].

The expression of CLas flagellins (*FlgA*) (DN3624_c0_g2_i1 and DN40834_c0_g1_i1) and several genes involved in the assembling and functioning of the flagellar machinery (*FliE, FliF*, *FliG*, *FliK, FliN, FliR, FliP, FlgB, FlgD, FlgE, FlgF, FlgG, FlgH, FlgI, FlgK, FlhA, FlhB, MotB,* and the *flagellar C-ring protein*) would indicate CLas assembles flagella for colonizing the gut of adult psyllids, although FlgF and FlgI were detected exclusively in *A3CLas*^*+*^. The expression of genes involved in the flagellar machinery corroborates recent data on the higher expression of genes encoding the flagellum apparatus of CLas in the gut of psyllids than in plants [87]. Although we detected a much higher number of transcripts of the flagellar system of the CLas isolate we worked with than those reported to be expressed in the gut of psyllids by Andrade et al. [87], we did not detect the expression of three genes they evaluated (*fliQ, fliL*, and *flgA*). Differences in the overall number of genes of the flagellar system among lineages are expected to occur, but not among isolates of the same species [88].

The production of flagellum in the gut by infecting CLas is also supported by transmission electron microscopy images, although most of the CLas cells observed lacked a flagellum [87]. Analysis of images of the flagellum of CLas cells clearly shows CLas carries the secondary flagellar system, producing a lateral flagellum. The secondary flagellar systems arose twice in the evolution of bacteria, once in alpha-proteobacteria and once in the common ancestor of beta/gamma-proteobacteria [89]. The primary flagellar system produces a polar flagellum and contributes with cell motility in liquid media, while the secondary flagellum system produces a lateral flagellum that is involved in adhesion and cell swarming on surfaces. Bacterial flagella serve bacteria not only as a motor apparatus, but also as a protein export/assembly apparatus. The motor apparatus of flagella can provide bacteria the movement required to remain in the gut, as demonstrated in the trypanosomatid *Vickermania* [90]. Moreover, the flagellum pattern can be altered by bacteria depending on the environmental conditions faced, and bacterial flagella are recognized as important virulence factors [91–93]. Chemotaxis is an important contribution of bacterial flagellum to virulence [94], and the detection of the expression of four chemotaxis protein genes (DN15199, DN31800, DN46197 and DN50630) suggests the flagellum participates in cell adhesion to and swarming on the psyllid gut epithelium. Yet, we also propose the flagellum aids CLas to become chemically oriented for the localization of suitable host cells for invasion.

Biofilm formation requires bacteria to synthesize an extracellular matrix. Biofilms include extracellular proteins, cell surface adhesins and subunits of flagella and pili proteins [95]. We did not identify the expression of adhesins in the transcriptome of CLas in the gut of adult psyllids. Nevertheless, several chaperones were highly expressed, and many chaperones can act as adhesins in bacteria. Additionally, we believe the cold shock protein (DN10086_c0_g2_i1) of CLas is also playing key roles in biofilm formation. Cold shock proteins were demonstrated not only to participate in biofilm formation but also to support cell adhesion and motility, and to stimulate cell aggregation, interfering thus with virulence [96, 97].

### Cross-talk for infecting the gut epithelium

Several gut-associated bacterial symbionts of hemipterans enter in close contact with epithelial cells to establish an intracellular phase through endocytosis [98]. CLas also enters the gut epithelium of psyllids through endocytosis, remaining inside vacuoles formed from or surrounded by endoplasmic reticulum membrane [99]. The differential gene expression analysis of the gut of *CLas*^*+*^ psyllids led to the identification of transcripts involved with ultrastructural alterations of the gut epithelial cells, allowing the identification of the molecular intermediates that participate in the route CLas follows to invade the gut epithelium.

In addition to the regulation of the immune related responses earlier described in the gut epithelium of *CLas*^*+*^ psyllids, epithelial cells of the gut were altered early in the infection process (*A1CLas*^*+*^ adults). Alterations are perceived by the transcriptional down-regulation of zonadhesin-like (DN14644_c0_g1_i1), a transcriptional response already reported by Ramsey et al. [100]. Zonadhesin-like proteins represent an expansion of the zonadhesin multi-domain proteins that are implicated in the binding of sperm and egg in a species-specific manner. In the gut, zonadhesin-like proteins are thought to function as mucins, and two zonadhesin-like proteins of *D. citri* were previously predicted to be part of the extracellular matrix and to have a role in cell-cell adhesion. Mucins protect the gut epithelium from microbial infections and inflammation [101]. Mucins are components of the perimicrovillar layer that in psyllids and other paraneopterans [102] replaces the chitin-based matrix (peritrophic membrane) that provides a protective barrier to the gut epithelial cells from pathogenic bacteria [103].

In vertebrates the mucin composition of the mucous layer can vary among gut regions, with diet composition and gut microbial infection [104]. Mucins can also be exploited as a nutritional resource by bacteria, and degradation of mucins by a commensal microbe was demonstrated to facilitate the penetration of the epithelium by viruses [105, 106]. The role different mucins can have on the host-pathogen interaction can explain the up-regulation of the psyllid gene coding for mucin-5AC (DN16207_c6_g5_i1) in *A3CLas*^*+*^ psyllids. The higher abundance of this protein in *CLas*^*+*^ psyllids has been demonstrated in previous proteomic analysis [107]. Regulation of the host mucin-5AC has been reported in vertebrate hosts infected by Gram^-^ and Gram^*+*^ bacteria, and in one system modulation of mucin-5AC has been linked with increased adhesion of bacteria to the gut tissue [108, 109].

The differential expression of genes coding for cytoskeletal proteins involved in the regulation of the submembranous actin-spectrin network in cells of the gut epithelium of *CLas*^*+*^ psyllids demonstrates CLas interferes with the remodeling of gut epithelial junctions in adult psyllids, leading to endocytosis of intercellular junctions with cellular organelles. Adducins (protein Hu-li tai shao - DN19744_c0_g2_i2) form connections with membranes, promote spectrin-actin interactions and regulate actin filaments [110]. Internalization of apical junction and/or tight junction proteins may disrupt the epithelial barriers and favor the movement of bacterial toxins into cells [111].

The intracellular events in preparation for endocytosis can also be detected as indicated by the up-regulation of the psyllid phosphatase 1 regulatory subunit 21 (DN19112_c0_g1_i1) in the gut of *A1CLas*^*+*^. This protein participates in early (sorting process) or late (maturation) endosome pathway, which leads to the endocytosis of several types of materials, including pathogenic agents. Our hypothesis is that early endosomes will also internalize the leptin receptor overlapping protein-like 1. The leptin receptor overlapping transcript-like 1 (DN20058_c2_g3_i4) was down-regulated in the gut epithelium of *A2CLas*^*+*^ psyllids. Internalization of such receptors in early endosomes also affects cell signaling, and in this particular case can severely interferes with the gut epithelium resistance to microbial infection. Down-regulation of leptin/leptin receptor was reported to drastically affect cell resistance to amoeba and bacterial infection in several species [112–114].

The endocytic vesicles detach and become free endocytic carrier vesicles transporting their cargoes to late endosomes to finally fuse with endoplasmic reticulum, where CLas cells aggregate in associated vacuoles as indicated by ultrastructural analysis [99]. Fusion of lysosomes to cell aggregates vacuoles is inhibited by the down-regulation of soluble NSF attachment protein (SNAPs) (DN21196_c2_g1_i1) in *A2CLas*^*+*^. SNAPs are highly conserved proteins that participate in intracellular membrane fusion and vesicular trafficking [115]. Intracellular membrane fusion requires both SNAPs and NSF to act in concert, and inhibition of one of them will lead to the failure of membrane fusion and the accumulation of vesicles one cannot fuse [116].

SNAPs are characterized by the presence of a tetratricopeptide repeat (TPR) domain [117]. Tetratricopeptide repeat proteins (TRP) are directly involved in virulence, particularly due the translocation of virulence factors into host cells and in the blockage of phagolysosomal maturation, among others [118]. Three TRPs (DN11192_c0_g1_i1; DN11192_c0_g1_i2; DN14826_c0_g1_i2) were expressed in the gut of *CLas*^*+*^ psyllids, but only DN14826_c0_g1_i2 was differentially expressed. The expression of different TRPs in all sampling periods and their time-specific differential expression indicates TRPs participates in different processes of CLas interactions with the host cells, from CLas establishment in the gut lumen to epithelial cell invasion and access to the hemocoel.

The down-regulation of the psyllids annexin B9-like isoform X1 (DN19784_c8_g1_i2) in the gut of *A3CLas*^*+*^ would interferes with the development of multivesicular bodies, once annexin B9 is involved in endosomal trafficking to multivesicular bodies [119]. This change will affect the transfer of the contents of CLas-containing endosomal vesicles to lysosomes for proteolytic degradation.

We propose that the establishment of the intracellular cycle and survival of CLas within host cells was putatively aided by the expression of CLas peptidyl-prolyl isomerase (PPI) (DN14386_c0_g1_i1), a protein that has been proved to participate in intracellular infection and virulence of other Gram^-^ bacteria [120–122]. The Mpi PPI from *Legionella pneumophila* was demonstrated to require an active enzymatic site to enhance a proper host cell invasion [123], although the preservation of the active site was not a requirement for the PPI of *Burkholderia pseudomallei* [120]. Ghanim et al. [99] suggested earlier that CLas cells do not enter the ER, but instead CLas cells recruit ER to transform the phagosome into a host-immune free space suitable for CLas survival and multiplication. These same ER-derived structures were already observed in *Legionella* and *Brucella* [124, 125].

Early endosomes and endoplasmic reticulum share contact sites that are used to bind to microtubules at or close to their contact sites, and both organelles remain bounded as endosomes traffic and mature [126]. Endosome trafficking within the cell involves the membrane binding to motor proteins and its transport along the actin and microtubule cytoskeleton [127]. Trafficking of endosomes in *CLas*^*+*^ psyllids is provided by the up-regulation of kinesin-like protein KIF3B isoform X4 (DN10161_c0_g1_i1) in the gut of psyllids. Kinesins are motor proteins involved in the movement of multiple cytoplasmic organelles [127].

Profilin (DN19422_c2_g2_i1), another protein involved in intracellular movement of organelles, was also up-regulated in the gut of *A2CLas*^*+*^ psyllids. Profilins are actin-binding proteins capable of regulating actin polymerization and the availability of the actin cytoskeleton for binding to the endosomes; but new roles for profilins are being identified in vertebrates [128]. Additionally, profilin 1 plays an important role in host–pathogen interactions. Intracellular pathogens such as *Listeria monocytogenes* and *Shigella flexneri* use the host-cell actin cytoskeleton to propel themselves through the cytoplasm and to spread to neighboring cells without entering the extracellular space [128, 129]. Dynein (DN35915_c0_g1_i1), another motor protein, was up-regulated in the gut of *A3CLas*^*+*^ psyllids. Dyneins also contribute to microtubule-based transport in eukaryotic cells [130–132].

### Cross-talk for moving to the hemocoel

After CLas inhibits the early immune response and invades the intracellular space of the gut epithelium by regulating a clathrin-independent endocytosis mechanism, hiding from the host’s immune activators within vacuoles surrounded by endoplasmic reticulum membrane, where CLas multiplies, the molecular mechanism behind the release of CLas bacteria from epithelial cells in the hemocoel may require additional data.

The higher expression in *A3CLas*^*+*^ of seven (DN15630_c0_g2_i3 = CLIBASIA_00255; DN15330_c0_g1_i2 = CLIBASIA_00880; DN14809_c0_g1_i = CLIBASIA_03170; DN14146_c0_g1_i1 = CLIBASIA = 04410; DN10141_c0_g1_i1 = CLIBASIA_00995; DN15141_c0_g1_i1 = CLIBASIA_01620; DN15414_c0_g1_i4 = CLIBASIA_01605) out of the 67 Sec-dependent proteins common to *Candidatus* Liberibacter species [133] indicates these candidate effector proteins play an important role in the dynamics of CLas infection of the psyllid gut epithelium at this stage. In fact, genomic analysis of Liberibacter species predicted a total of 166 proteins containing Sec-dependent signal peptides in the CLas strain psy62, from which 86 have been already experimentally validated [134]. These are potential effector proteins, and 106 of them share common homologues with Las_Ishi-1 and Las_gxpsy. But only 45 Sec-dependent proteins were shared among HLB-associated *Ca*. Liberibacter species (CLas, CLam and CLaf) [135, 136]. The detection of transcripts of the general secretion system provides further support for the participation of the detected Sec-dependent proteins in the late process of infection of the gut epithelium of *D. citri*. The Sec pathway secretion system transports proteins involved in bacterial cell functions and survival [133]. The Sec pathway is represented by two independent pathways, both detected in the gut of *CLas*^*+*^ psyllids: the post-translational pathway represented by the expression of SecA (DN14962_c0_g1_i1) and SecY (DN15568_c0_g3_i3); and the co-translational pathway represented by the signal recognition particle receptor FtsY (DN1686_c0_g1_i1) [137]. We did not detect SecB expression in the gut of adult psyllids, indicating SecA can also act as a chaperone complementing the required activity of SecB as reported in *Escherichia coli* [138].

Combined ultrastructure, proteomics and transcriptomics analysis of the gut epithelium of CLas-infected psyllids suggested CLas acquisition into the hemocoel would rely on the programmed cell death of CLas-infected epithelial gut cells, although CLas infection has little impact on the fitness of adult psyllids [19]. The low fitness impact of CLas infection to adult *D. citri* indicates CLas acquisition into the hemocoel would occur through an exocytosis process, as suggested for the *Ca.* Liberibacter solanacearum vectored by potato psyllids [139].

By following the proposed subroutines based on mechanistic and essential aspects of cell death proposed by the Nomenclature Committee on Cell Death [140], our transcriptomic analysis could not support the proposition that CLas-infected epithelial gut cells of psyllids would initiate the process of programmed cell death (PCD) as proposed by Kruse et al. [19]. We did not observe transcription of caspases, a very common protein to several subroutines of cell death, as well as other aspects that would allow this characterization [140]. Yet, we were unable to confidently characterize one of the subroutines of cell death described when analyzing the transcriptional profile of *CLas*^*+*^ psyllids, once the molecular mechanisms involved were not all represented. However, we can confidently report the activation/inhibition of the expression of genes involved in processes of regulated cell death. The high expression of the lysosomal cathepsin L1 (DN15698_c4_g11_i2) in *A2CLas*^*+*^ psyllids would support the activation of lysosome-dependent cell death, which requires intracellular perturbations to permeabilize the lysosomal membrane to result in the release of cytosolic cathepsins. Mitochondrial outer membrane permeabilization and caspases are not necessarily required for this process of cell death, and lysosome-dependent cell death is an important response to pathophysiological conditions induced by intracellular pathogens [140].

The overexpression of the psyllid BCL2/adenovirus E1B 19 kDa protein-interacting protein 3 (BNIP3) (DN_20168_c2_g3_i4) in *A3CLas*^*+*^ also supports the gut epithelium of *CLas*^*+*^ psyllids go into lysosome-dependent cell death. BNIP3 are pro-apoptotic proteins that open the pores of mitochondrial outer membrane, resulting in mitochondria dysregulation due the loss of membrane potential and ROS production in mitochondria [141, 142]. But BNIP3 oligomerization with BCL2 has been demonstarted not to be required for cell death [141]. Besides, the down-regulation of the psyllid senescence-associated protein transcript (DN16041_c1_g1_i2) in the gut of *A3CLas*^*+*^ psyllids demonstrates CLas suppresses cell senescence and the further dysfunctional growth of the epithelial cells due the activation of senescence-associated secretory phenotype [143, 144]. Cellular senescence is not considered a form of regulated cell death [140].

The differential expression of a putative juvenile hormone binding protein (JHBP) (DN21423_c1_g12_i2; DN21423_c1_g12_i1) in *CLas*^*+*^ psyllids (down-regulation in the gut of *A2CLas*^*+*^; up-regulation in *A3CLas*^*+*^) demonstrates the epithelial cells count with different levels of hormonal stimulation. JHBPs are proteins that act as shuttles for juvenile hormone (JH) in the hemolymph, avoiding JH degradation by JH-esterases and JH-epoxide hydrolases [145]. JH is produced and released primarily by neurosecretory cells of the *corpora allata* of the central nervous system, and the availability of JHBP in the gut, if its activity in the gut is the same played in the hemolymph, indicates JH is alo available in the gut epithelial cells. JH has recently been shown to be produced by intestinal stem cells and enteroblasts of the gut epithelium of *D. melanogaster,* and to regulate cell growth and survival. The local JH activity was also shown relevant for damage response by gut cells, playing important roles in gut homeostasis [146]. If the pattern of expression of JHBP correlates with the availability of JH we can argue that at the same time CLas acts on the regulation of the gut epithelium by suppressing cell senescence, it can also induce the proliferation of stem cells in order to replace cells that were damaged due the release of CLas cell into the hemocoel and/or entered the lysosome-mediated cell death as discussed earlier.

The observed up-regulation of the transcriptional levels of nocturnin (DN19927_c10_g1_i6) in the gut of *A2CLas*^*+*^ psyllids suggests that this circadian rhythm effector protein may be acting together with JH in regulating gut genes, just as the joint action of the circadian genes and JH in the regulation of genes acting on the transitional states of *Pyrrhocoris apterus* adults to reproductive diapause or not [147]. Nocturnin is classified as a deadenylase acting on the catalysis of poly(A) tail of target mRNAs and/or targeting noncoding RNAs, and has been implicated in metabolic regulation, development and differentiation [148]. But recent studies with *curled,* the nocturnin ortholog in *Drosophila* proved nocturnin is an NADP(H)2’-phosphatase acting on the conversion of the dinucleotide NADP^*+*^ into NAD^*+*^ and NADPH into NADH, regulating mitochondrial activity and cellular metabolism in response to circadian clock [149]. NADP(H)2’-phosphatase activity of nocturnin was later reported in vertebrates, with the demonstration of the colocalization of nocturnin in mitochondria, cytosol and endoplasmic reticulum-bound pools depending on the isoform [150]. Thus, nocturnin acts as a regulator of the intracellular levels of NADP(H) and the oxidative stress response. The role of nocturnin in the regulation of cell metabolism and the oxidative stress response supports the required energy supply to sustain the increased gene expression activity in *CLas*^*+*^ gut epithelium cells.

The expression profile of CLas infecting the gut epithelium of *A3CLas*^*+*^ psyllids demonstrates CLas has an increased protein synthesis activity as expected by the overexpression of DEAD/DEAH box helicase (DN4052_c0_g1_i1), a protein involved in ribosome biogenesis, RNA turnover and translation initiation [151]. Increased protein activity is also supported by the increased expression of molecular chaperones, such as the trigger factor (DN13945_c0_g1_i1), GroEL (DN14757_c0_g1_i1) and DnaK (DN15220_c0_g1_i1), which prevent protein misfolding and aggregation [152, 153].

### CLas multiprotein complexes

The protein HlyD family efflux transporter periplasmic adaptor subunit (DN11766_c0_g1_i1) and the outer membrane factor translocation protein TolB (DN15087_c0_g1_i3) are essential components for functioning the pump of tripartite efflux systems. HlyD connects primary and secondary inner membrane transporters to the outer membrane factor TolB. CLas expressed several inner membrane transporters belonging to the MSF and ABC families. TolB has been shown to interact with a range of proteins including cell-killing proteins [154, 155]. In *Xylella fastidiosa* TolB was shown to be involved in biofilm development as one of the members of the Tol – Pal system. TolB associates with other proteins than Pal in the outer membrane and periplasm, aiding *X. fastidiosa* in maintaining membrane integrity [156].

### Additional CLas transcriptional regulators

The detection of several other uncharacterized response regulators (DN10585_c0_g1_i1; DN10585_c0_g2_i1; DN12200_c0_g1_i1; DN58285_c0_g1_i1) and transcriptional regulators (DN11066_c0_g1_i1; DN15145_c0_g1_i2; DN15145_c0_g1_i5) indicates other CLas two-component systems are also being activated by environmental stimuli, such as the two-component system sensor histidine kinase AtoS (DN14305_c0_g2_i1; DN15701_c0_g1_i1). The two-component system sensor histidine kinase AtoS is a member of the AtoS/AtoC regulatory system, but we did not identify the AtoC response regulator in the CLas transcriptome. This regulatory system is better known by the induction of AtoS by acetoacetate. AtoS then phosphorylates AtoC that will in turn stimulate the expression of the atoDAEB operon for the catabolism of short chain fatty acids. We could not reliably identify transcripts belonging to the *atoDAEB* operon to demonstrate CLas requirements for short chain fatty acids. We propose that the activation of the AtoS/AtoC regulatory system is instead acting on the regulation of the flagellar regulon, regulating the expression of CLas genes involved in cell motility and chemotaxis, as reported for *Escherichia coli* in response to acetoacetate or spermidine [157].

### Cross-talk on nutrient deficiency

The high expression of sulfonate ABC transporter permease (DN15690_c0_g1_i2) in all *CLas*^*+*^ psyllids suggests CLas signals the host to supply its requirements for sulfur. Sulfur is a ubiquitous element involved in a number of different processes in organisms [158]. The sulfonate ABC transporter permease is involved in sulfur/sulfonate uptake, and the higher expression observed in *A3CLas*^+^ as compared to *A1CLas*^+^ points for an increased demand late in the infection process.

Sulfur is also used in the biogenesis of Fe-S clusters, which are produced in conditions of oxidative stress and iron deprivation. The biogenesis of Fe-S clusters was observed by the expression of two unclassified cysteine sulfurases (DN12917_c0_g1_i1 and DN35541_c0_g1_i1), the cysteine desulfuration protein SufE (DN1144_c0_g1_i1), the Fe-S cluster assembly protein SulfB (DN14252_c0_g1_i1), and the iron-sulfur cluster carrier protein ApbC (DN17762_c4_g2_i3) [159, 160]. All four genes had an increased expression in *A3CLas*^+^ as compared to *A1CLas*^+^. Sulfur requirements by CLas could also be used for protection against oxidative stress as demonstrated by the expression of the thioredoxin-dependent thiol peroxidase (DN10526_c0_g1_i1) [161, 162].

The high expression of CLas phosphate ABC transporter permease subunit PstC (DN40256_c0_g1_i1; DN67283_c0_g1_i1), putative two-component sensor histidine kinase transcriptional regulatory protein (DN14305_c0_g2_i1), two-component sensor histidine kinase (PhoR) (DN28075_c0_g1_i1; DN67626_c0_g1_i1), two component response regulator protein (PhoB) (DN14869_c0_g1_i1), alkaline phosphatase (DN12828_c0_g1_i2; DN39297_c0_g1_i2) and NTP pyrophosphohydrolase (DN2128_c0_g1_i1) demonstrates CLas cells are exposed to phosphate restriction while infecting the gut epithelium of adult psyllids. Phosphate is generally the major source of phosphorus to bacteria, a vital nutrient for living organisms. Phosphorus is important in several processes (energy metabolism, intracellular signaling, among others). Misregulation of phosphorus availability in bacteria is sensed by the PhoB/PhoR two-component regulatory system, leading to the activation of the Pho regulon. The Pho regulon regulates the expression of other genes, producing phenotypes that can differ in morphology and virulence in response to phosphate deprivation [163, 164]. Thus, the expression of both members of the PhoB/PhoR two component regulatory system and the phosphate ABC transporter permease subunit PstC, that is in charge to transport extracellular phosphate to the cytosol, proves CLas was exposed to phosphate concentrations below 4 μM, a threshold that generally turns on the expression of the response regulator PhoB. Furthermore, the expression of alkaline phosphatase and NTP pyrophosphohydrolase, which both act on phosphorus-containing substrates [163] demonstrates CLas increased the catabolism of substrates capable of releasing phosphorus nutrient.

The requirement of CLas for phosphate is also demonstrated by the up-regulation of the psyllid glycerophosphocholine phosphodiesterase GPCPD1 gene, as observed by the increased abundance of its transcript (DN15741_c0_g2_i2) in *A3CLas*^*+*^. The contribution of GPCPD1 in phosphate production results from the downstream processing of GPCPD1 hydrolysis products of glycerophosphocholine, choline and glycerolphosphate. At the same time glycerophosphocholine hydrolysis can provide phosphates for cell metabolism, the choline can serve as as substrate for phosphatidylcholine synthesis. Phosphatidylcholine is a major lipid component of membranes of eukaryotes, although several bacterial symbionts and pathogens carry phosphatidylcholine synthases, showing their requirement for this lipid component of cell membranes, including those belonging to *Rhizobiaceae* as *Ca*. Liberibacter. Choline serves as substrate for the osmoprotectant glycine betaine synthesis and as an energy substrate to support cell growth in *Rhizobiaceae* [165, 166].

The role of host-produced choline in CLas metabolism is supported by the up-regulation of CLas glycine/betaine ABC transporter substrate-binding protein (DN15178_c0_g1_i1) in the gut of *A3CLas*^*+*^. ABC transporters were reported to have several important roles in *Ca.* Liberibacter asiaticus, from the importation of nutrients like choline to the exportation of virulence factors [167]. Thus, we believe the increased activity of GPCPD1 in *A3CLas*^*+*^ is a result of the recycling of glycerophosphocholine for choline utilization in the recovery of membranes of the gut epithelial cells of the host psyllid as a response to cell infection and/or in the supplementation of choline to CLas metabolism.

CLas is known to induce profound physiological changes in citrus plants, affecting the nutritional content of CLas-infected plants to psyllids, which have in turn altered transcriptional profiles and protein abundance when feeding on CLas-infected and health citrus plants [24, 107]. Therefore, it is impossible to distinguish if the indication of nutritional restriction CLas encounters when infecting the gut epithelium of psyllids is an indirect effect of the host plant on the psyllids or if this is a direct response of psyllids to avoid CLas infection.

## Conclusion

We conclude that CLas basically express its full arsenal of genes early in the invasion process of the gut of adults of *D. citri*. CLas survival in the gut of psyllids involves the regulation of ROS production and availability in the psyllid gut, and consequently the inhibition of activation ROS-dependent nitric oxide production and nitric oxide-dependent antimicrobial peptide synthesis. Our data suggests that gene expression regulation of adult psyllids is based on the up-regulation of proteins involved in alternative splice site selection. CLas regulates the peristalsis of the gut of the host and highly expresses genes involved in adherence proteins and biofilm formation to colonize the gut lumen. Our analysis also indicates the gut epithelial cells are invaded by CLas by a process mediated by the down-regulation of leptin receptor and its internalization in early endosomes in the psyllid gut cells. This process is followed by the regulation of several host genes that lead to vesicles containing CLas, which includes the suppression of the cell machinery involved in the process of cell senescence. The intense cross talk between CLas and the host cells is mediated by a high number of candidate effector proteins we identified. These proteins are potential targets for the development of management strategies directed to interfere with essential metabolic pathways to invasion, infection and multiplication of CLas in the vector *D. citri*.

## Material and Methods

### *Diaphorina citri* rearing and plants

A colony of CLas-free Asian Citrus Psyllid (ACP) was initiated with insects collected from *Murraya paniculata* (L.) Jack, syn. *Murraya exotica* L. (Sapindales: Rutaceae) in the state of São Paulo, Brazil in 2009.

### *Diaphorina citri* gut collection and RNA extraction

Adults of *D. citri* (7 to 10 days after emergence) were transferred to uninfected and CLas-infected citrus plants [*Citrus* x *sinensis* (L.) Osbeck, grafted in ‘Rangpur’ lime (*C*. x *limonia* Osbeck)] for an exposure period (EP) of 1, 2, 3, 4, 5 and 6 days. For each exposure period, three biological replicates were collected (1 replicate = 100 individuals). Third-instars of *D. citri* were also transferred to CLas-infected and uninfected (control) citrus plants for collection of RNA and differential transcriptional analysis. Nymphs were allowed to feed for 4 days on CLas-infected and control plants. Afterwards, three biological replicates/treatment (1 replicate = 200 nymphs) were collected and stored in RNALater (Invitrogen/ThermoFisher Scientific, Waltham, MA, United States, USA) for further processing and analysis.

After each exposure time, adults were collected and the gut dissected under aseptic conditions. A similar procedure was used for nymphs, but in this case insects we opted by extracting RNA from whole nymphs, as initial attempts to dissect the suitable gut samples for downstream analysis were very time-consuming and had a low success rate.

The obtained guts/nymphs were stored in RNALater (Invitrogen/ThermoFisher Scientific, Waltham, MA, USA) at -80°C until RNA extraction. Total RNA extraction was performed using the SV RNA Isolation System Kit (Promega Madison, WI, USA), following the manufacturers’ recommendations. Samples were lysed in 175 μL of RNA lysis buffer added with β-mercaptoethanol and 350 μL of RNA dilution buffer for tissue disruption using a TissueLyser II LT^TM^ (Qiagen, Hilden, Germany). Samples were incubated at 70°C for 3 min, centrifuged (12,000 *g* × 10 min × 4°C), trapped in a column and washed with 95% ethanol by centrifugation (12,000 *g* × 10 min × 4°C). Total RNA was recovered in 350 μL of RNA wash solution following centrifugation (12,000 *g* × 1 min × 4°C). Afterwards, samples were treated with 50 μL of DNAse mix (40 μL buffer ‘yellow core’+ 5 μL 0.09 M MnCl_2_ + 5 μL DNAse I) for 15 min at room temperature. Samples were added with 200 μL of DNAse stop solution and subjected to centrifugation (13,000 *g* × 1 min × 4°C). Samples were washed in 600 μL cleaning solution followed by a second wash in 250 μL following centrifugation (12,000 *g* × 1 min × 4°C). The pelleted RNA was recovered in 30 μL of nuclease-free water, and RNA concentration verified using NanoDrop V 3.8.1 (ThermoFischer).

RNA samples containing residual DNA contaminants were further treated with 2 μL of buffer (10×) and 2 μL of Turbo^TM^ DNAse (2 U/μl) (Ambion/ThermoFisher Scientific, Vilnius Lithuania). Samples were incubated at 37°C for 30 min followed by DNAse inactivation by adding 2 μL of DNAse Inactivation Reagent (Ambion/ThermoFisher Scientific, Vilnius Lithuania). After 5 min at room temperature, samples were centrifuged (13,000 *g* × 1.5 min × 4°C) and the supernatant collected and stored at -80°C. DNA elimination was confirmed by testing the amplification of the wingless gene (*wg*) of ACP [168].

All plants and insects were subjected to quantitative PCR analysis for verification of CLas infection using the TaqMan qPCR Master Mix Kit (Ambion /ThermoFisher Scientific, Vilnius Lithuania) [169]. In insects, the average Ct value found for the *wg* gene in the libraries was 30.7, while the average Ct value in plants for the 16S rRNA gene from CLas was 29.6. Samples with a Ct value under 35 were considered positive for CLas.

After confirmation of the *Ca.* L. asiaticus infection status of each sample, RNA obtained from the gut samples of adults were pooled in equimolar concentrations to yield three exposure periods: *i) A1CLas*^*-*^: adults that fed on healthy citrus plant (CLas^-^) for 1-2 days; *ii) A2CLas*^*-*^: for 3-4 days; *iii) A3CLas*^*-*^: for 5-6 days; *iv) A1CLas*^*+*^: adults that fed on infected citrus plant (CLas^*+*^) for 1-2 days; *v) A2CLas*^*+*^: for 3-4 days; and *vi) A3CLas*^*+*^: for 5-6 days. In the case of the whole nymphs, two samples were produced: *N1CLas*^*+*^: for nymphs after 4 days of feeding on CLas-infected citrus plants, and *N1CLas*^*-*^: for nymphs after 4 days of feeding on control plants.

Samples were subjected to mRNA enrichment through eukaryote and prokaryote rRNA removal using the Ribo-Zero rRNA Removal Epidemiology Kit (Illumina, San Diego, CA, USA), following the manufacturers’ instructions. RNA integrity was confirmed using the Agilent Bioanalyzer 1000 (Agilent Technologies, Santa Clara, CA, USA).

### Library preparation and sequencing

cDNA libraries were prepared for sequencing using the cDNA TruSeq RNA Library Prep Kit (Illumina, San Diego, CA, USA) following a paired-end (2 × 100 bp) strategy. The cDNA produced was end-repaired and adenosine was added at the 3′ end of each cDNA fragment to guide the ligation of specific adapters. Adapters consisted of primers for transcription and a specific index to code each sample. Samples were enriched with limited-cycle PCR and analyzed to confirm the success of sample preparation before sequencing using the Illumina HiScanSQ platform (Illumina, San Diego, CA, USA) available at the Multiusers Center of Agricultural Biotechnology at the Department of Animal Sciences, ESALQ/USP.

### *De novo* transcriptome assembly

Reads quality were visualized in FastQC software [170] before adapters removal and quality filtering by trimming the leading (LEADING:3) and trailing (TRAILING:3) nucleotides until the quality was higher than 3, and then using a sliding window of 4 nucleotides and trimming when scores were lower than 22 (SLIDINGWINDOW 4:22). Quality filtering was done using Trimmomatic-0.36 [171].

All reads obtained were used to assemble a *de novo* transcriptome using the pipeline available in the Trinity-v.2.4.0 software [172]. Both paired and unpaired trimmed and quality-filtered reads were used to assemble the *de novo* transcriptome, which was further used as the reference transcriptome for the RNA-Seq experiments. Assemblage was obtained using normalization of the reads coverage (<50) and the minimum contig size selected was 200 nucleotides.

The transcripts obtained were functionally annotated using the BlastX algorithm for putative identification of homologous sequences, with an *e*-value cut-off < 10^–3^. Annotated sequences were curated and grouped into categories according to their function using Blast2Go® [173] with an *e*-value cut-off < 10^–6^ and EggNOG-mapper 4.5.1 [174]. Transcripts putatively identified as belonging to insects and *Ca.* L. asiaticus were checked against the KEGG database (Kyoto Encyclopedia of Genes and Genomes) [175] to verify the metabolic pathways represented in the obtained *de novo* transcriptome. Transcripts of *Diaphorina citri*, *Ca.* Liberibacter spp. and *Wolbachia* spp. were filtered using Blast2Go version Pro [176].

### Differential gene expression analyses

Changes in the pattern of gene expression were evaluated separately in gut of ACP adults infected or not by *Ca*. L. asiaticus using the CLC Genomics Workbench 20.0 software (QIAGEN, Aarhus, Denmark). Reads from each library were counted against the *de novo* transcriptome, and counts were normalized as transcripts per million reads (TPM). TPM values for each sample were used to calculate fold-change ratios for comparative analyses of the gene expression of control (CLas^-^) versus CLas-infected insects (CLas^*+*^) within each feeding interval (1-2 d, 3-4 d, 5-6 d). Only the transcript that were counted in two of the replicates of a particular treatment were further taken for comparative analysis. Data were analyzed using multifactorial statistics based on a negative binomial Generalized Linear Model (GLM). The values of fold change obtained were corrected with False Discovery Rate (FDR) method and only transcripts that showed *p*-value ≤0.05 and log fold change > |2| between treatments were considered differentially expressed.

## Supplementary Information

**Additional file 1:** The analysis of the 248,850 contigs was conducted by Blast2Go, using Blastx to recover annotations with significant homology from the NCBI. All terms ‘Biological Processes’, ‘Molecular Function’ and ‘Cellular Component’ at level 2 are represented as percent over total number of sequences.

**Additional file 2: **Distribution of alignments and transcripts of *de novo* assembly of *Diaphorina citri* gut that fed on health and CLas-infected citrus plant, after search for similarity in NCBI databank.

**Additional file 3: **Differentially expressed CLas genes in the gut of *Diaphorina citri* adults after 1-2 d (*A1CLas+*), 3-4 d (*A2CLas+*) and 5-6 d (*A3CLas+*) of infection by feeding on CLas-infected citrus plants.

## Data Availability

All cDNA libraries produced from RNA obtained from samples analyzed are fully available as SRA files through NCBI (https://www.ncbi.nlm.nih.gov/bioproject/) under BioProject ID PRJNA722422. All remaining data generated or analysed during this study are included in this published article and its additional information files.

## Abbreviations

*A1CLas*: adults that fed on healthy citrus plant for 1-2 days
*A2CLas*: adults that fed on healthy citrus plant for 3-4 days
*A3CLas*^*-*^: adults that fed on healthy citrus plant for 5-6 days
*A1CLas*^*+*^: adults of Asian Citrus Psyllid that fed on CLas-infected citrus plant for 1-2 days
*A2CLas*^*+*^: adults of Asian Citrus Psyllid that fed on CLas-infected citrus plant for 3-4 days
*A3CLas*^*+*^: adults of Asian Citrus Psyllid that fed on CLas-infected citrus plant for 5-6 days
ACP: Asian Citrus Psyllid
ADK: Adipokinetic Hormone
AMP: Antimicrobial Peptides
BLAST: Basic Local Alignment Search Tool
BNIP3: BCL2/Adenovirus E1B 19 Kda Protein-Interacting Protein 3
CLaf: *Candidatus* Liberibacter africanus
CLam: *Candidatus* Liberibacter americanus
CLas: *Candidatus* Liberibacter asiaticus
*CLas*^*-*^: adults of Asian Citrus Psyllid that fed on healthy citrus plant
*CLas*^*+*^: adults of Asian Citrus Psyllid that fed on CLas-infected citrus plant
DE: Differential Expression
ER: Endoplasmic Reticulum
FC: Fold Change
FDR: False Discovery Rate
GLM: Generalized Linear Model
HLB: Huanglongbing
JH: Juvenile Hormone
JHBP: Juvenile Hormone Binding Protein
KEGG: Kyoto Encyclopedia of Genes and Genomes
*N1CLas*^*-*^: Nymphs of Asian Citrus Psyllid fed for 4 days on healthy citrus plants
*N1CLas*^*+*^: Nymphs of Asian Citrus Psyllid fed for 4 days of feeding on CLas-infected citrus plants
PhoB: Two-component Response Regulator Protein
PhoR: Two-component Sensor Histidine Kinase
PPI: Peptidyl-Prolyl Isomerase
PPO: Prophenoloxidases
RBOH: Respiratory Burst Oxidase Homologs
RNS: Reactive Nitrogen Species
ROS: Reactive Oxygen Species
SNAP: Soluble NSF Attachment Protein
TPM: Transcripts Per Million Reads
TPR: Tetratricopeptide Repeat

## References

[1] Huang W, Reyes-Caldas P, Mann M, Seifbarghi S, Kahn A, Almeida RPP (2020). Bacterial vector-borne plant diseases: unanswered questions and future directions. Mol Plant..

[2] Perilla-Henao LM, Casteel CL (2016). Vector-borne bacterial plant pathogens: interactions with hemipteran insects and plants. Front Plant Sci..

[3] Mauck KE, Chesnais Q, Shapiro LR (2018). Evolutionary determinants of host and vector manipulation by plant viruses. Adv Virus Res..

[4] Bové JM (2006). Huanglongbing: A destructive newly emerging, century-old disease of citrus. J Plant Pathol..

[5] Graça JV, Douhan GW, Halbert SE, Keremane ML, Lee RF, Vidalakis G, Zhao H (2016). Huanglongbing: An overview of a complex pathosystem ravaging the world's citrus. J Integr Plant Biol..

[6] Tomaseto AF, Marques RN, Fereres A, Zanardi OZ, Volpe HXL, Alquézar B, Peña L, Miranda MP (2019). Orange jasmine as a trap crop to control *Diaphorina citri*. Sci Rep..

[7] Bassanezi RB, Lopes AS, Miranda MP, Wulff NA, Volpe HXL, Ayres AJ (2020). Overview of citrus huanglongbing spread and management strategies in Brazil. Trop Plant Pathol..

[8] Abebe E, Gugsa G, Ahmed M. Review on major food-borne zoonotic bacterial pathogens. J Trop Med. 2020;4674235. 10.1155/2020/4674235. 10.1155/2020/4674235 PMC7341400 32684938

[9] Dala-Paula BM, Plotto A, Bai J, Manthey JA, Baldwin EA, Ferrarezi RS, Gloria MBA (2019). Effect of huanglongbing or greening disease on orange juice quality, a review. Front Plant Sci..

[10] Singerman A, Rogers ME. The economic challenges of dealing with citrus greening: the case of Florida. J Integr Pest Manag. 2020;11(1):1-7. doi: 10.1093/jipm/pmz037.

[11] CABI/EPPO. *Candidatus* Liberibacter asiaticus [Distribution map]. 4th ed. Wallingford: CABI.; 2017.

[12] Ajene IJ, Khamis FM, van Asch B, Pietersen G, Seid N, Rwomushana I (2020). Distribution of *Candidatus* Liberibacter species in Eastern Africa, and the first report of *Candidatus* Liberibacter asiaticus in Kenya. Sci Rep..

[13] Wulff NA, Daniel B, Sassi RS, Moreira AS, Bassanezi RB, Sala I (2020). Incidence of *Diaphorina citri* carrying *Candidatus* Liberibacter asiaticus in Brazil's citrus belt. Insects..

[14] Jagoueix S, Bove JM, Garnier M (1994). The phloem-limited bacterium of greening disease of citrus is a member of the alpha subdivision of the Proteobacteria. Int J Syst Bacteriol..

[15] Bové JM, Garnier M (2003). Phloem-and xylem-restricted plant pathogenic bacteria. Plant Sci..

[16] Bendix C, Lewis JD (2018). The enemy within: phloem-limited pathogens. Mol Plant Pathol..

[17] Ammar E-D, Shatters RG, Hall DG (2011). Localization of *Candidatus* Liberibacter asiaticus, associated with citrus Huanglongbing disease, in its psyllid vector using fluorescence in situ hybridization. J Phytopathol..

[18] Ammar E-D, Hall DG, Shatters RG (2017). Ultrastructure of the salivary glands, alimentary canal and bacteria-like organisms in the Asian citrus psyllid, vector of citrus Huanglongbing disease bacteria. J Microsc Ultrastruct..

[19] Kruse A, Fattah-Hosseini S, Saha S, Johnson R, Warwick E, Sturgeon K (2017). Combining 'omics and microscopy to visualize interactions between the Asian citrus psyllid vector and the Huanglongbing pathogen *Candidatus* Liberibacter asiaticus in the insect gut. PLoS ONE..

[20] Ammar E-D, Achor D, Levy A (2019). Immuno-ultrastructural localization and putative multiplication sites of Huanglongbing bacterium in Asian Citrus Psyllid *Diaphorina citri*. Insects..

[21] Gray S, Cilia M, Ghanim M (2014). Circulative, "nonpropagative" virus transmission: an orchestra of virus-, insect-, and plant-derived instruments. Adv Virus Res..

[22] Ammar E-D, Ramos JE, Hall DG, Dawson WO, Shatters RG (2016). Acquisition, replication and inoculation of *Candidatus* Liberibacter asiaticus following various acquisition periods on Huanglongbing-infected citrus by nymphs and adults of the Asian Citrus Psyllid. PLoS ONE..

[23] Martinelli AF, Uratsu SL, Albrecht U, Reagan RL, Phu MP, Britton M (2012). Transcriptome profiling of citrus fruit response to Huanglongbing disease. PLoS ONE..

[24] Fu S, Shao J, Zhou C, Hartung JS (2016). Transcriptome analysis of sweet orange trees infected with *'Candidatus* Liberibacter asiaticus' and two strains of Citrus Tristeza Virus. BMC Genomics..

[25] Hu Y, Zhong X, Liu X, Lou B, Zhou C, Wang X (2017). Comparative transcriptome analysis unveils the tolerance mechanisms of *Citrus hystrix* in response to ‘*Candidatus* Liberibacter asiaticus’ infection. PLoS ONE..

[26] Ammar E, Shatters RG Jr, Lynch C, Hall DG. Detection and relative titer of *Candidatus* Liberibacter asiaticus in the salivary glands and alimentary canal of *Diaphorina citri* (Hemiptera: Psyllidae) vector of citrus Huanglongbing disease. Ann Entomol Soc Am. 2011;104(3):526–33. 10.1603/AN10134.

[27] Ammar E-D, George J, Sturgeon K, Stelinski LL, Shatters RG (2020). Asian citrus psyllid adults inoculate Huanglongbing bacterium more efficiently than nymphs when this bacterium is acquired by early instar nymphs. Sci Rep.

[28] Vyas M, Fisher TW, He R, Nelson W, Yin G, Cicero JM, et al. Asian citrus psyllid expression profiles suggest *Candidatus* Liberibacter asiaticus-mediated alteration of adult nutrition and metabolism, and of nymphal development and immunity. PLoS ONE. 2015;10(6):e0130328. 10.1371/journal.pone.0130328. 10.1371/journal.pone.0130328 PMC4474670 26091106

[29] Yu HZ, Li NY, Zeng XD, Song JC, Yu XD, Su HN (2020). Transcriptome analyses of *Diaphorina citri* midgut responses to *Candidatus* Liberibacter asiaticus infection. Insects..

[30] Liu K, He J, Guan Z, Zhong M, Pang R, Han Q (2021). Transcriptomic and metabolomic analyses of *Diaphorina citri* Kuwayama infected and non-infected with *Candidatus* Liberibacter asiaticus. Front Physiol.

[31] Molki B, Thi Ha P, Mohamed A, Killiny N, Gang DR, Omsland A, Beyenal H (2019). Physiochemical changes mediated by "*Candidatus* Liberibacter asiaticus" in Asian citrus psyllids. Sci Rep..

[32] Santos-Ortega Y, Killiny N (2018). Silencing of sucrose hydrolase causes nymph mortality and disturbs adult osmotic homeostasis in *Diaphorina citri* (Hemiptera: Liviidae). Insect Biochem Mol Biol..

[33] Lu ZJ, Huang YL, Yu HZ, Li NY, Xie YX, Zhang Q (2019). Silencing of the chitin synthase gene is lethal to the Asian citrus psyllid *Diaphorina citri*. Int J Mol Sci..

[34] Lu ZJ, Zhou CH, Yu HZ, Huang YL, Liu YX, Xie YX (2019). Potential roles of insect tropomyosin1-X1 isoform in the process of *Candidatus* Liberibacter asiaticus infection of *Diaphorina citri*. J Insect Physiol..

[35] Yu X, Killiny N (2020). RNA interference-mediated control of Asian citrus psyllid, the vector of the Huanglongbing bacterial pathogen. Trop Plant Pathol..

[36] Lehane MJ, Billingsley PF. Biology of the insect midgut. Dordrecht: Springer Netherlands; 1996. 10.1007/978-94-009-1519-0.

[37] Erlandson MA, Toprak U, Hegedus DD (2019). Role of the peritrophic matrix in insect-pathogen interactions. J Insect Physiol..

[38] Buchon N, Broderick NA, Lemaitre B (2013). Gut homeostasis in a microbial world: insights from *Drosophila melanogaster*. Nat Rev Microbiol..

[39] Vallet-Gely I, Lemaitre B, Boccard F (2008). Bacterial strategies to overcome insect defences. Nat Rev Microbiol..

[40] Perkins A, Nelson KJ, Parsonage D, Poole LB, Karplus PA (2015). Peroxiredoxins: guardians against oxidative stress and modulators of peroxide signaling. Trends Biochem Sci..

[41] Knoops B, Argyropoulou V, Becker S, Ferté L, Kuznetsova O (2016). Multiple roles of peroxiredoxins in inflammation. Mol Cells.

[42] Nelson KJ, Knutson ST, Soito L, Klomsiri C, Poole LB, Fetrow JS (2011). Analysis of the peroxiredoxin family: using active-site structure and sequence information for global classification and residue analysis. Proteins..

[43] Jain M, Munoz-Bodnar A, Zhang S, Gabriel DW (2018). A secreted *'Candidatus* Liberibacter asiaticus' peroxiredoxin simultaneously suppresses both localized and systemic innate immune responses in planta. Mol Plant Microbe Interact..

[44] Kodrík D, Bednářová A, Zemanová M, Krishnan N (2015). Hormonal regulation of response to oxidative stress in insects-an update. Int J Mol Sci..

[45] Turner AJ, Isaac RE, Coates D (2001). The neprilysin (NEP) family of zinc metalloendopeptidases: genomics and function. Bioessays..

[46] Shears SB, Hayakawa Y (2019). Functional multiplicity of an insect cytokine family assists defense against environmental stress. Front Physiol..

[47] Neish AS (2013). Redox signaling mediated by the gut microbiota. Free Radic Res..

[48] Wu SC, Liao CW, Pan RL, Juang JL (2012). Infection-induced intestinal oxidative stress triggers organ-to-organ immunological communication in *Drosophila*. Cell Host Microbe..

[49] Jing T, Wang F, Qi F, Wang Z (2018). Insect anal droplets contain diverse proteins related to gut homeostasis. BMC Genomics..

[50] Melcarne C, Lemaitre B, Kurant E (2019). Phagocytosis in *Drosophila*: From molecules and cellular machinery to physiology. Insect Biochem Mol Biol..

[51] Anderson KV (2000). Toll signaling pathways in the innate immune response. Curr Opin Immunol..

[52] Nakhleh J, Moussawi LE, Osta MA (2017). The melanization response in insect immunity. Adv In Insect Phys..

[53] Stokes BA, Yadav S, Shokal U, Smith LC, Eleftherianos I (2015). Bacterial and fungal pattern recognition receptors in homologous innate signaling pathways of insects and mammals. Front Microbiol..

[54] Brunner E, Peter O, Schweizer L, Basler K (1997). *pangolin* encodes a Lef-1 homologue that acts downstream of *armadillo* to transduce the wingless signal in *Drosophila*. Nature..

[55] Franz A, Shlyueva D, Brunner E, Stark A, Basler K (2017). Probing the canonicity of the Wnt/Wingless signaling pathway. PLoS Genet..

[56] Strand M, Micchelli CA (2011). Quiescent gastric stem cells maintain the adult *Drosophila* stomach. Proc Natl Acad Sci U S A..

[57] Cordero JB, Stefanatos RK, Scopelliti A, Vidal M, Sansom OJ (2012). Inducible progenitor-derived Wingless regulates adult midgut regeneration in *Drosophila*. EMBO J..

[58] Riddell CE, Lobaton Garces JD, Adams S, Barribeau SM, Twell D, Mallon EB (2014). Differential gene expression and alternative splicing in insect immune specificity. BMC Genomics..

[59] Girard C, Will CL, Peng J, Makarov EM, Kastner B, Lemm I (2012). Post-transcriptional spliceosomes are retained in nuclear speckles until splicing completion. Nat Commun..

[60] Galganski L, Urbanek MO, Krzyzosiak WJ (2017). Nuclear speckles: molecular organization, biological function and role in disease. Nucleic Acids Res..

[61] Bhattacharyya S, Feferman L, Tobacman JK (2014). Arylsulfatase B regulates versican expression by galectin-3 and AP-1 mediated transcriptional effects. Oncogene..

[62] García B, Merayo-Lloves J, Martin C, Alcalde I, Quirós LM, Vazquez F (2016). Surface Proteoglycans as mediators in bacterial pathogens infections. Front Microbiol..

[63] Roberts IS (1996). The biochemistry and genetics of capsular polysaccharide production in bacteria. Annu Rev Microbiol..

[64] Zhao M, Wang S, Li F, Dong D, Wu B (2016). arylsulfatase b mediates the sulfonation-transport interplay in human embryonic kidney 293 cells overexpressing sulfotransferase 1A3. Drug Metab Dispos..

[65] Massenti R, Lo Bianco R, Sandhu AK, Gu L, Sims C (2016). Huanglongbing modifies quality components and flavonoid content of 'Valencia' oranges. J Sci Food Agric..

[66] Dala-Paula BM, Raithore S, Manthey JA, Baldwin EA, Zhao W (2018). Active taste compounds in juice from oranges symptomatic for Huanglongbing (HLB) citrus greening disease. LWT – Food Science Technology..

[67] Kiefl J, Kohlenberg B, Hartmann A, Obst K, Paetz S, Krammer G, Trautzsch S (2018). Investigation on key molecules of Huanglongbing (HLB)-induced orange juice off-flavor. J Agric Food Chem..

[68] Treutter D (2006). Significance of flavonoids in plant resistance: a review. Environ Chem Lett..

[69] Hijaz FM, Manthey JA, Folimonova SY, Davis CL, Jones SE, Reyes-De-Corcuera JI (2013). An HPLC-MS characterization of the changes in sweet orange leaf metabolite profile following infection by the bacterial pathogen *Candidatus* Liberibacter asiaticus. PLoS ONE..

[70] Hijaz F, Al-Rimawi F, Manthey JA, Killiny N (2020). Phenolics, flavonoids and antioxidant capacities in *Citrus* species with different degree of tolerance to Huanglongbing. Plant Signal Behav..

[71] Li Y, Park JS, Deng JH, Bai Y (2006). Cytochrome c oxidase subunit IV is essential for assembly and respiratory function of the enzyme complex. J Bioenerg Biomembr..

[72] Benguettat O, Jneid R, Soltys J, Loudhaief R, Brun-Barale A, Osman D, Gallet A (2018). The DH31/CGRP enteroendocrine peptide triggers intestinal contractions favoring the elimination of opportunistic bacteria. PLoS Pathog..

[73] Hammer TJ, Janzen DH, Hallwachs W, Jaffe SP, Fierer N (2017). Caterpillars lack a resident gut microbiome. Proc Natl Acad Sci U S A..

[74] Wegener C, Veenstra JA (2015). Chemical identity, function and regulation of enteroendocrine peptides in insects. Curr Opin Insect Sci..

[75] Caccia S, Casartelli M, Tettamanti G (2019). The amazing complexity of insect midgut cells: types, peculiarities, and functions. Cell Tissue Res..

[76] Wu K, Li S, Wang J, Ni Y, Huang W, Liu Q, et al. Peptide hormones in the insect midgut. Front Physiol. 2020;11:191. 10.3389/fphys.2020.00191. 10.3389/fphys.2020.00191 PMC7066369 32194442

[77] Panz M, Vitos-Faleato J, Jendretzki A, Heinisch JJ, Paululat A, Meyer H. A novel role for the non-catalytic intracellular domain of neprilysins in muscle physiology. Biol Cell. 2012;104(9):553–68. 10.1111/boc.201100069. 10.1111/boc.201100069 22583317

[78] Klein DC (2007). Arylalkylamine N-acetyltransferase: "the Timezyme". J Biol Chem..

[79] Hiragaki S, Suzuki T, Mohamed AA, Takeda M (2015). Structures and functions of insect arylalkylamine N-acetyltransferase (iaaNAT); a key enzyme for physiological and behavioral switch in arthropods. Front Physiol..

[80] Amherd R, Hintermann E, Walz D, Affolter M, Meyer UA (2000). Purification, cloning, and characterization of a second arylalkylamine N-acetyltransferase from *Drosophila melanogaster*. DNA Cell Biol..

[81] Brodbeck D, Amherd R, Callaerts P, Hintermann E, Meyer UA, Affolter M (1998). Molecular and biochemical characterization of the aaNAT1 (Dat) locus in *Drosophila melanogaster*: differential expression of two gene products. DNA Cell Biol..

[82] Solari P, Rivelli N, De Rose F, Picciau L, Murru L, Stoffolano JG, Liscia A (2017). Opposite effects of 5-HT/AKH and octopamine on the crop contractions in adult *Drosophila melanogaster*: Evidence of a double brain-gut serotonergic circuitry. PLoS ONE..

[83] Zhang B, Gong J, Zhang W, Xiao R, Liu J, Xu XZS (2018). Brain-gut communications via distinct neuroendocrine signals bidirectionally regulate longevity in *C. elegans*. Genes Dev..

[84] Giltner CL, Nguyen Y, Burrows LL (2012). Type IV pilin proteins: versatile molecular modules. Microbiol Mol Biol Rev..

[85] Kazmierczak BI, Schniederberend M, Jain R (2015). Cross-regulation of *Pseudomonas* motility systems: the intimate relationship between flagella, pili and virulence. Curr Opin Microbiol..

[86] Andrade M, Wang N (2019). The Tad Pilus apparatus of ‘*Candidatus* Liberibacter asiaticus' and its regulation by VisNR. Mol Plant Microbe Interact..

[87] Andrade MO, Pang Z, Achor DS, Wang H, Yao T, Singer BH, Wang N (2020). The flagella of ‘*Candidatus* Liberibacter asiaticus’ and its movement *in planta*. Mol Plant Pathol..

[88] Bardy SL, Ng SYM, Jarrell KF (2003). Prokaryotic motility structures. Microbiology..

[89] Liu R, Ochman H (2007). Origins of flagellar gene operons and secondary flagellar systems. J Bacteriol..

[90] Kostygov AY, Frolov AO, Malysheva MN, Ganyukova AI, Chistyakova LV, Tashyreva D, et al. *Vickermania* gen. nov., trypanosomatids that use two joined flagella to resist midgut peristaltic flow within the fly host. BMC Biol. 2020;18(1):187. 10.1186/s12915-020-00916-y. 10.1186/s12915-020-00916-y PMC7712620 33267865

[91] Moens S, Vanderleyden J (1996). Functions of bacterial flagella. Crit Rev Microbiol..

[92] Macnab RM (2003). How bacteria assemble flagella. Annu Rev Microbiol..

[93] Duan Q, Zhou M, Zhu L, Zhu G (2013). Flagella and bacterial pathogenicity. J Basic Microbiol..

[94] Matilla MA, Krell T. The effect of bacterial chemotaxis on host infection and pathogenicity. FEMS Microbiol Rev. 2018;42(1). 10.1093/femsre/fux052. 10.1093/femsre/fux052 29069367

[95] Fong JNC, Yildiz FH. Biofilm matrix proteins. Microbiol Spectr. 2015;3(2):10.1128/microbiolspec. MB-0004-2014. 10.1128/microbiolspec. MB-0004-2014. 10.1128/microbiolspec.MB-0004-2014 PMC4480581 26104709

[96] Eshwar AK, Guldimann C, Oevermann A, Tasara T (2017). Cold-shock domain family proteins (Csps) are involved in regulation of virulence, cellular aggregation, and flagella-based motility in *Listeria* monocytogenes. Front Cell Infect Microbiol..

[97] Ray S, Da Costa R, Thakur S, Nandi D (2020). *Salmonella* Typhimurium encoded cold shock protein E is essential for motility and biofilm formation. Microbiology..

[98] Nardi JB, Miller LA, Bee CM (2019). Interfaces between microbes and membranes of host epithelial cells in hemipteran midguts. J Morphol..

[99] Ghanim M, Achor D, Ghosh S, Kontsedalov S, Lebedev G, Levy A. *'Candidatus* Liberibacter asiaticus' accumulates inside endoplasmic reticulum associated vacuoles in the gut cells of *Diaphorina citri*. Sci Rep. 2017;7(1):16945. 10.1038/s41598-017-16095-w. 10.1038/s41598-017-16095-w PMC5717136 29208900

[100] Ramsey JS, Johnson RS, Hoki JS, Kruse A, Mahoney J, Hilf ME (2015). Metabolic interplay between the asian citrus psyllid and its *Profftella* symbiont: an Achilles' heel of the citrus greening insect vector. PLoS ONE..

[101] Hansson GC (2012). Role of mucus layers in gut infection and inflammation. Curr Opin Microbiol..

[102] Silva CP, Silva JR, Vasconcelos FF, Petretski MD, Damatta RA, Ribeiro AF, Terra WR (2004). Occurrence of midgut perimicrovillar membranes in paraneopteran insect orders with comments on their function and evolutionary significance. Arthropod Struct Dev..

[103] Kuraishi T, Binggeli O, Opota O, Buchon N, Lemaitre B (2011). Genetic evidence for a protective role of the peritrophic matrix against intestinal bacterial infection in *Drosophila melanogaster*. Proc Natl Acad Sci U S A..

[104] Paone P, Cani PD (2020). Mucus barrier, mucins and gut microbiota: the expected slimy partners?. Gut..

[105] Schroeder BO (2019). Fight them or feed them: how the intestinal mucus layer manages the gut microbiota. Gastroenterol Rep (Oxf)..

[106] Wu P, Sun P, Nie K, Zhu Y, Shi M, Xiao C, et al. A gut commensal bacterium promotes mosquito permissiveness to arboviruses. Cell Host Microbe. 2019;25(1):101-112.e5. 10.1016/j.chom.2018.11.004. 10.1016/j.chom.2018.11.004 30595552

[107] Ramsey JS, Chavez JD, Johnson R, Hosseinzadeh S, Mahoney JE, Mohr JP (2017). Protein interaction networks at the host-microbe interface in *Diaphorina citri*, the insect vector of the citrus greening pathogen. R Soc Open Sci..

[108] Dohrman A, Miyata S, Gallup M, Li JD, Chapelin C, Coste A (1998). Mucin gene (MUC 2 and MUC 5AC) upregulation by Gram-positive and Gram-negative bacteria. Biochim Biophys Acta..

[109] Quintana-Hayashi MP, Mahu M, De Pauw N, Boyen F, Pasmans F, Martel A (2015). The levels of *Brachyspira hyodysenteriae* binding to porcine colonic mucins differ between individuals, and binding is increased to mucins from infected pigs with *de novo* MUC5AC synthesis. Infect Immun..

[110] Matsuoka Y, Li X, Bennett V (2000). Adducin: structure, function and regulation. Cell Mol Life Sci..

[111] Hopkins AM, Walsh SV, Verkade P, Boquet P, Nusrat A (2003). Constitutive activation of Rho proteins by CNF-1 influences tight junction structure and epithelial barrier function. J Cell Sci..

[112] Faggioni R, Feingold KR, Grunfeld C (2001). Leptin regulation of the immune response and the immunodeficiency of malnutrition. FASEB J..

[113] Guo X, Roberts MR, Becker SM, Podd B, Zhang Y, Chua SC (2011). Leptin signaling in intestinal epithelium mediates resistance to enteric infection by *Entamoeba histolytica*. Mucosal Immunol..

[114] Mackey-Lawrence NM, Petri WA (2012). Leptin and mucosal immunity. Mucosal Immunol..

[115] Stenbeck G (1998). Soluble NSF-attachment proteins. Int J Biochem Cell Biol..

[116] Rothman JE (1994). Mechanisms of intracellular protein transport. Nature..

[117] Lakhssassi N, Liu S, Bekal S, Zhou Z, Colantonio V, Lambert K (2017). Characterization of the soluble NSF attachment protein gene family identifies two members involved in additive resistance to a plant pathogen. Sci Rep..

[118] Cerveny L, Straskova A, Dankova V, Hartlova A, Ceckova M, Staud F, Stulik J (2013). Tetratricopeptide repeat motifs in the world of bacterial pathogens: role in virulence mechanisms. Infect Immun..

[119] Tjota M, Lee SK, Wu J, Williams JA, Khanna MR, Thomas GH (2011). Annexin B9 binds to β(H)-spectrin and is required for multivesicular body function in *Drosophila*. J Cell Sci..

[120] Norville IH, Breitbach K, Eske-Pogodda K, Harmer NJ, Sarkar-Tyson M, Titball RW, Steinmetz I (2011). A novel FK-506-binding-like protein that lacks peptidyl-prolyl isomerase activity is involved in intracellular infection and in vivo virulence of *Burkholderia pseudomallei*. Microbiology..

[121] Pandey S, Tripathi D, Khubaib M, Kumar A, Sheikh JA, Sumanlatha G, Ehtesham NZ, Hasnain SE (2017). *Mycobacterium tuberculosis* peptidyl-prolyl isomerases are immunogenic, alter cytokine profile and aid in intracellular survival. Front Cell Infect Microbiol..

[122] Rasch J, Ünal CM, Klages A, Karsli Ü, Heinsohn N, Brouwer RMHJ (2018). Peptidyl-prolyl-cis/trans-isomerases Mip and PpiB of *Legionella pneumophila* contribute to surface translocation, growth at suboptimal temperature, and infection. Infect Immun..

[123] Helbig JH, König B, Knospe H, Bubert B, Yu C, Lück CP (2003). The PPIase active site of *Legionella pneumophila* Mip protein is involved in the infection of eukaryotic host cells. Biol Chem..

[124] Celli J, Gorvel JP (2004). Organelle robbery: *Brucella* interactions with the endoplasmic reticulum. Curr Opin Microbiol..

[125] Robinson CG, Roy CR (2006). Attachment and fusion of endoplasmic reticulum with vacuoles containing *Legionella pneumophila*. Cell Microbiol..

[126] Friedman JR, Dibenedetto JR, West M, Rowland AA, Voeltz GK (2013). Endoplasmic reticulum-endosome contact increases as endosomes traffic and mature. Mol Biol Cell..

[127] Granger E, McNee G, Allan V, Woodman P (2014). The role of the cytoskeleton and molecular motors in endosomal dynamics. Semin Cell Dev Biol..

[128] Witke W (2004). The role of profilin complexes in cell motility and other cellular processes. Trends Cell Biol..

[129] Kocks C (1994). Intracellular motility. Profilin puts pathogens on the actin drive. Curr Biol..

[130] Holzbaur EL, Vallee RB (1994). Dyneins: molecular structure and cellular function. Annu Rev Cell Biol..

[131] Porter ME (1996). Axonemal dyneins: assembly, organization, and regulation. Curr Opin Cell Biol..

[132] Hirokawa N (1998). Kinesin and dynein superfamily proteins and the mechanism of organelle transport. Science..

[133] Thapa SP, De Francesco A, Trinh J, Gurung FB, Pang Z, Vidalakis G, Wang N, Ancona V, Ma W, Coaker G (2020). Genome-wide analyses of Liberibacter species provides insights into evolution, phylogenetic relationships, and virulence factors. Mol Plant Pathol..

[134] Prasad S, Xu J, Zhang Y, Wang N (2016). SEC-Translocon dependent extracytoplasmic proteins of *Candidatus* Liberibacter asiaticus. Front Microbiol..

[135] Wang N, Pierson EA, Setubal JC, Xu J, Levy JG, Zhang Y, Li J, Rangel LT, Martins J (2017). The *Candidatus* Liberibacter-host interface: insights into pathogenesis mechanisms and disease control. Annu Rev Phytopathol..

[136] Andrade M, Li JY, Wang N (2020). *Candidatus* Liberibacter asiaticus: virulence traits and control strategies. Trop Plant Pathol..

[137] Green ER, Mecsas J. Bacterial secretion systems: an overview. Microbiol Spectr. 2016;4(1):10.1128/microbiolspec. VMBF-0012-2015. 10.1128/microbiolspec. VMBF-0012-2015. 10.1128/microbiolspec.VMBF-0012-2015 PMC4804464 26999395

[138] McFarland L, Francetić O, Kumamoto CA (1993). A mutation of *Escherichia coli* SecA protein that partially compensates for the absence of SecB. J Bacteriol..

[139] Cicero JM, Fisher TW, Qureshi JA, Stansly PA, Brown JK (2017). Colonization and intrusive invasion of potato psyllid by '*Candidatus* Liberibacter solanacearum'. Phytopathology..

[140] Galluzzi L, Vitale I, Aaronson SA, Abrams JM, Adam D, Agostinis P, Alnemri ES (2018). Molecular mechanisms of cell death: recommendations of the Nomenclature Committee on Cell Death 2018. Cell Death Differ..

[141] Vande Velde C, Cizeau J, Dubik D, Alimonti J, Brown T, Israels S (2000). BNIP3 and genetic control of necrosis-like cell death through the mitochondrial permeability transition pore. Mol Cell Biol..

[142] Moy RH, Cherry S (2013). Antimicrobial autophagy: a conserved innate immune response in *Drosophila*. J Innate Immun..

[143] Münch D, Amdam GV, Wolschin F (2008). Ageing in a eusocial insect: molecular and physiological characteristics of life span plasticity in the honey bee. Funct Ecol..

[144] Ito T, Igaki T (2016). Dissecting cellular senescence and SASP in *Drosophila*. Inflamm Regen..

[145] Zalewska M, Kochman A, Estève JP, Lopez F, Chaoui K (2009). Juvenile hormone binding protein traffic - Interaction with ATP synthase and lipid transfer proteins. Biochim Biophys Acta..

[146] Rahman MM, Franch-Marro X, Maestro JL, Martin D, Casali A (2017). Local juvenile hormone activity regulates gut homeostasis and tumor growth in adult *Drosophila*. Sci Rep..

[147] Bajgar A, Jindra M, Dolezel D (2013). Autonomous regulation of the insect gut by circadian genes acting downstream of juvenile hormone signaling. Proc Natl Acad Sci U S A..

[148] Hughes KL, Abshire ET, Goldstrohm AC (2018). Regulatory roles of vertebrate nocturnin: insights and remaining mysteries. RNA Biol..

[149] Estrella MA, Du J, Chen L, Rath S, Prangley E (2019). The metabolites NADP+ and NADPH are the targets of the circadian protein nocturnin (Curled). Nat Commun..

[150] Laothamatas I, Gao P, Wickramaratne A, Quintanilla CG, Dino A (2020). Spatiotemporal regulation of NADP(H) phosphatase nocturnin and its role in oxidative stress response. Proc Natl Acad Sci U S A..

[151] Redder P, Hausmann S, Khemici V, Yasrebi H, Linder P (2015). Bacterial versatility requires DEAD-box RNA helicases. FEMS Microbiol Rev..

[152] Agashe VR, Guha S, Chang HC, Genevaux P, Hayer-Hartl M, Stemp M (2004). Function of trigger factor and DnaK in multidomain protein folding: increase in yield at the expense of folding speed. Cell..

[153] Merz F, Hoffmann A, Rutkowska A, Zachmann-Brand B, Bukau B, Deuerling E (2006). The C-terminal domain of *Escherichia coli* trigger factor represents the central module of its chaperone activity. J Biol Chem..

[154] Carr S, Penfold CN, Bamfold V, James R, Hemmings AM (2000). The structure of TolB, an essential component of the tol-dependent translocation system, and its protein–protein interaction with the translocation domain of colicin E9. Structure..

[155] Loftus SR, Walker D, Maté MJ, Bonsor DA, James R, Moore GR, Kleanthous C (2006). Competitive recruitment of the periplasmic translocation portal TolB by a natively disordered domain of colicin E9. Proc Natl Acad Sci U S A..

[156] Santos CA, Janissen R, Toledo MA, Beloti LL, Azzoni AR (1854). Characterization of the TolB-Pal trans-envelope complex from *Xylella fastidiosa* reveals a dynamic and coordinated protein expression profile during the biofilm development process. Biochim Biophys Acta..

[157] Theodorou MC, Theodorou EC, Kyriakidis DA (2012). Involvement of AtoSC two-component system in *Escherichia coli* flagellar regulon. Amino Acids..

[158] Beinert H (2000). A tribute to sulfur. Eur J Biochem..

[159] Layer G, Gaddam SA, Ayala-Castro CN, Ollagnier-de Choudens S, Lascoux D (2007). SufE transfers sulfur from SufS to SufB for iron-sulfur cluster assembly. J Biol Chem..

[160] Roche B, Aussel L, Ezraty B, Mandin P, Py B, Barras F (2013). Iron/sulfur proteins biogenesis in prokaryotes: formation, regulation and diversity. Biochim Biophys Acta..

[161] Lu J, Holmgren A (2014). The thioredoxin antioxidant system. Free Radic Biol Med..

[162] Wang C, Chen Y, Zhou H, Li X, Tan Z (2020). Adaptation mechanisms of *Rhodococcus* sp. CNS16 under different temperature gradients: Physiological and transcriptome. Chemosphere..

[163] Lamarche MG, Wanner BL, Crépin S, Harel J (2008). The phosphate regulon and bacterial virulence: a regulatory network connecting phosphate homeostasis and pathogenesis. FEMS Microbiol Rev..

[164] Santos-Beneit F (2015). The Pho regulon: a huge regulatory network in bacteria. Front Microbiol..

[165] Sohlenkamp C, López-Lara IM, Geiger O (2003). Biosynthesis of phosphatidylcholine in bacteria. Prog Lipid Res..

[166] Dupont L, Garcia I, Poggi MC, Alloing G, Mandon K, Le Rudulier D (2004). The *Sinorhizobium meliloti* ABC transporter Cho is highly specific for choline and expressed in bacteroids from *Medicago sativa* nodules. J Bacteriol..

[167] Li W, Cong Q, Pei J, Kinch LN, Grishin NV (2012). The ABC transporters in *Candidatus* Liberibacter asiaticus. Proteins..

[168] Manjunath KL, Halbert SE, Ramadugu C, Webb S, Lee RF (2008). Detection of *'Candidatus* Liberibacter asiaticus' in *Diaphorina citri* and its importance in the management of citrus huanglongbing in Florida. Phytopathology..

[169] Li W, Hartung JS, Levy L (2006). Quantitative real-time PCR for detection and identification of *Candidatus* Liberibacter species associated with citrus huanglongbing. J Microbiol Methods..

[170] Andrews S. FastQC: a quality control tool for high throughput sequence data [software]. 2010 [cited 2021 Feb 26]. Available from: http://www.bioinformatics.babraham.ac.uk/projects/fastqc.

[171] Bolger AM, Lohse M, Usadel B (2014). Trimmomatic: a flexible trimmer for Illumina sequence data. Bioinformatics..

[172] Haas BJ, Papanicolaou A, Yassour M, Grabherr M, Blood PD, Bowden J (2013). *De novo* transcript sequence reconstruction from RNA-seq using the Trinity platform for reference generation and analysis. Nat Protoc..

[173] Conesa A, Götz S, García-Gómez JM, Terol J, Talón M, Robles M (2005). Blast2GO: a universal tool for annotation, visualization and analysis in functional genomics research. Bioinformatics..

[174] Huerta-Cepas J, Forslund K, Coelho LP, Szklarczyk D, Jensen LJ, von Mering C, Bork P (2017). Fast genome-wide functional annotation through orthology assignment by eggNOG-Mapper. Mol Biol Evol..

[175] Kanehisa M, Goto S (2000). KEGG: Kyoto encyclopedia of genes and genomes. Nucleic Acids Res..

[176] Götz S, García-Gómez JM, Terol J, Williams TD, Nagaraj SH, Nueda MJ (2008). High-throughput functional annotation and data mining with the Blast2GO suite. Nucleic Acids Res..

