# Supplementary material for: The molecular interplay of the establishment of an infection – gene expression of Diaphorina citri gut and Candidatus Liberibacter asiaticus
Source: BMC Genomics. 2021 Sep 21;22:677. doi: 10.1186/s12864-021-07988-2 (PMC8454146; doi:10.1186/s12864-021-07988-2)
Supplement: Supplementary file 1 — Additional file 1: The analysis of the 248,850 contigs was conducted by Blast2Go, using Blastx to recover annotations with significant homology from the NCBI. All terms ‘Biological Processes’, ‘Molecular Function’ and ‘Cellular Component’ at level 2 are represented as percent over total number of sequences. [file 12864_2021_7988_MOESM1_ESM.pdf]

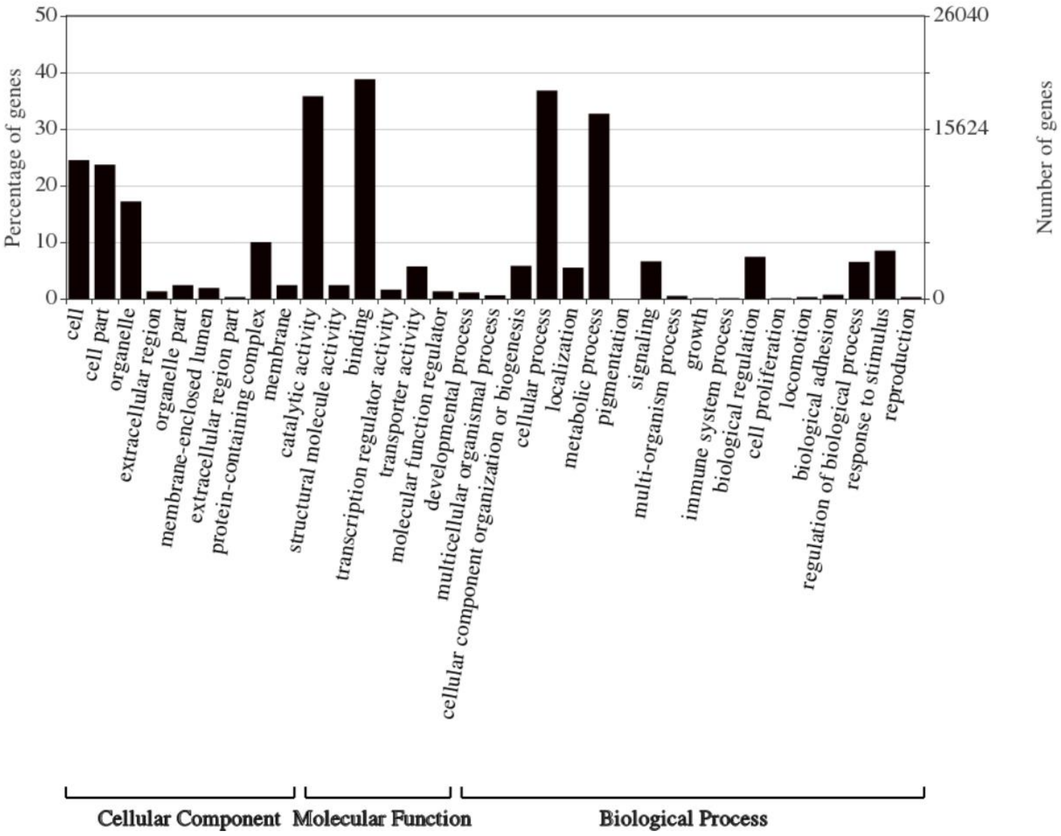

Additional file 1. The analysis of the 248,850 contigs was conducted by Blast2Go, using Blastx to recover annotations with significant homology from the NCBI. All terms ‘Biological Processes’, ‘Molecular Function’ and ‘Cellular Component’ at level 2 are represented as percent over total number of sequences.
