# Supplementary material for: The molecular interplay of the establishment of an infection – gene expression of Diaphorina citri gut and Candidatus Liberibacter asiaticus
Source: BMC Genomics. 2021 Sep 21;22:677. doi: 10.1186/s12864-021-07988-2 (PMC8454146; doi:10.1186/s12864-021-07988-2)
Supplement: Supplementary file 2 — Additional file 2: Distribution of alignments and transcripts of de novo assembly of Diaphorina citri gut that fed on health and CLas-infected citrus plant, after search for similarity in NCBI databank. [file 12864_2021_7988_MOESM2_ESM.pdf]

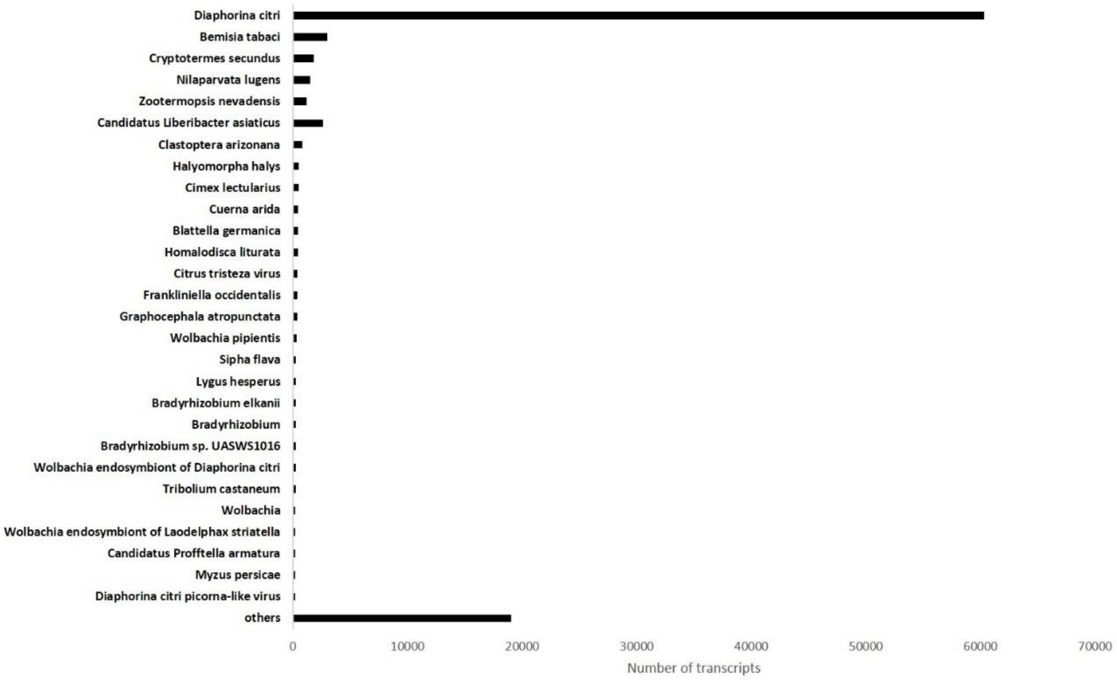

Additional file 2. Distribution of alignments and transcripts of *de novo* assembly of *Diaphorina citri* gut that fed on health and CLas-infected citrus plant, after search for similarity in NCBI databank.
