# Supplementary material for: The molecular interplay of the establishment of an infection – gene expression of Diaphorina citri gut and Candidatus Liberibacter asiaticus
Source: BMC Genomics. 2021 Sep 21;22:677. doi: 10.1186/s12864-021-07988-2 (PMC8454146; doi:10.1186/s12864-021-07988-2)
Supplement: Supplementary file 3 — Additional file 3: Differentially expressed CLas genes in the gut of Diaphorina citri adults after 1-2 d (A1CLas+), 3-4 d (A2CLas+) and 5-6 d (A3CLas+) of infection by feeding on CLas-infected citrus plants. [file 12864_2021_7988_MOESM3_ESM.pdf]

Additional file 3. Differentially expressed CLas genes in the gut of *Diaphorina citri* adults after 1-2 d (*A1CLas*$^{+}$), 3-4 d (*A2CLas*$^{+}$) and 5-6 d (*A3CLas*$^{+}$) of infection by feeding on CLas-infected citrus plants.

Comparison	Annotation	Transcript ID	FC	FDR
***A1CLas+ x *****A3CLas+**	2-octaprenyl-6-methoxyphenyl hydroxylase	DN14876_c0_g1_i1	13.9	0.02
	5-(carboxyamino)imidazole ribonucleotide synthase	DN14087_c0_g1_i2	12.6	0.03
	50S ribosomal protein L2	DN11710_c0_g1_i2	16.9	0.04
	AAA family ATPase	DN1679_c0_g1_i1	13.8	0.05
	alanine--tRNA ligase	DN14954_c0_g1_i1	17.2	0.01
	alpha/beta hydrolase	DN39212_c0_g1_i1	14.0	0.01
	amino acid ABC transporter permease	DN15630_c0_g2_i3	14.1	0.02
	argininosuccinate lyase	DN13884_c0_g1_i1	13.4	0.01
	aspartate aminotransferase	DN14989_c0_g1_i4	12.0	0.02
	aspartate aminotransferase family protein	DN11157_c0_g1_i1	13.9	0.03
	ATP-dependent protease ATPase subunit HslU	DN20210_c0_g3_i1	12.0	0.03
	beta-ketoacyl-ACP synthase I	DN14910_c0_g1_i1	13.8	0.04
	bifunctional glutamate N-acetyltransferase/amino-acid acetyltransferase ArgJ	DN10638_c0_g1_i1	14.6	0.03
	carbamoyl phosphate synthase small subunit	DN13724_c0_g1_i1	19.0	$9.3e^{-3}$
	Chloramphenicol-sensitive protein rarD	DN13473_c0_g1_i1	17.2	0.05
	chromosomal replication initiation protein	DN15038_c0_g1_i2	16.8	$7.41e^{-3}$
	cold-shock protein	DN10086_c0_g2_i1	20.0	0.02
	DEAD/DEAH box helicase	DN4052_c0_g1_i1	24.2	0.02
	DNA polymerase III subunit chi	DN12825_c0_g1_i1	11.2	0.04
	DNA topoisomerase I	DN14080_c0_g1_i1	11.9	0.04
	DNA topoisomerase IV subunit B	DN10717_c0_g1_i1	37.8	$8.87e^{-4}$
	DNA translocase FtsK	DN14450_c0_g1_i1	22.7	$2.49e^{-3}$
	DUF1874 domain-containing protein	DN14305_c0_g1_i1	31.4	$9.46e^{-4}$
	elongation factor G	DN17043_c0_g1_i1	14.2	0.05
	elongation factor P	DN12683_c0_g1_i1	31.9	0.01
	elongation factor Ts	DN14637_c0_g1_i1	11.1	0.04
	excinuclease ABC subunit C	DN45886_c0_g1_i1	14.8	0.03
	exodeoxyribonuclease VII large subunit	DN17192_c0_g4_i3	14.0	$9.3e^{-3}$
	exonuclease I	DN15585_c7_g1_i1	11.2	0.02
	fumarate hydratase	DN14954_c0_g1_i2	23.9	$6.8e^{-3}$
	glutaminase	DN14270_c0_g1_i1	13.0	0.03
	glutathione S-transferase	DN4544_c0_g1_i1	17.5	0.04
	glycerol kinase	DN16960_c2_g1_i14	34.4	0.01
	glycine/betaine ABC transporter substrate-binding protein	DN15178_c0_g1_i1	33.1	$2.07e^{-3}$
	hypothetical protein	DN14113_c1_g1_i1	16.2	0.02

hypothetical protein	DN17456_c0_g1_i2	23.7	0.01
hypothetical protein	DN17456_c0_g1_i3	14.2	0.03
hypothetical protein	DN17456_c0_g1_i4	22.0	3.66e^{-3}
hypothetical protein	DN17456_c0_g2_i1	17.3	0.04
hypothetical protein CGUJ_01660	DN14113_c0_g2_i1	16.5	0.05
hypothetical protein DJ66_0664	DN16265_c0_g2_i2	30.5	1.15e^{-3}
IMP dehydrogenase	DN6964_c0_g2_i1	13.6	0.03
isocitrate dehydrogenase	DN15328_c0_g1_i1	13.5	0.06
leucyl aminopeptidase	DN13997_c0_g1_i1	34.9	0.02
LuxR family transcriptional regulator	DN14940_c0_g2_i1	11.9	0.05
molecular chaperone DnaK	DN15220_c0_g1_i1	28.2	0.01
molecular chaperone GroEL	DN14757_c0_g1_i1	20.2	0.03
N-acetyl-gamma-glutamyl-phosphate reductase	DN15330_c0_g1_i2	14.7	0.05
NADH dehydrogenase	DN15210_c0_g1_i2	14.4	0.03
NifU family protein	DN5715_c0_g1_i1	11.8	0.05
nitrate ABC transporter ATP-binding protein	DN14488_c0_g2_i1	12.8	0.03
orotidine 5'-phosphate decarboxylase/BAX inhibitor (BI)-1/YccA family protein	DN14873_c0_g1_i1	13.5	0.04
PAS domain-containing sensor histidine kinase	DN14346_c0_g1_i1	13.9	0.02
penicillin-binding protein	DN14783_c0_g1_i1	11.9	0.03
periplasmic solute binding protein	DN15099_c0_g1_i1	9.7	0.04
permease	DN13997_c0_g2_i1	14.8	0.03
peroxiredoxin	DN17546_c4_g1_i1	13.5	0.03
phage repressor protein	DN15458_c0_g2_i5	20.6	0.03
phosphopyruvate hydratase	DN14337_c0_g1_i1	9.4	0.03
phosphoribosylformylglycinamidine cyclo-ligase	DN27217_c0_g1_i1	17.8	4.73e^{-3}
pilus assembly protein	DN13708_c0_g1_i2	10.8	0.03
poly(A) polymerase	DN12871_c0_g1_i1	11.9	0.05
porin	DN10141_c0_g1_i1	86.2	5.55e^{-4}
porin family protein	DN15690_c0_g1_i2	19.8	0.03
putative protease IV transmembrane protein	DN14809_c0_g1_i2	12.7	0.05
pyridoxine 5'-phosphate synthase	DN15414_c0_g1_i4	19.1	0.01
pyruvate kinase/carbamoyl phosphate synthase large subunit	DN15141_c0_g1_i1	15.8	0.04
replicative DNA helicase	DN15002_c0_g1_i2	20.9	0.03
response regulator	DN12200_c0_g1_i1	16.8	0.02
ribonuclease D	DN10298_c0_g1_i1	16.4	0.03
signal recognition particle protein	DN15243_c0_g1_i2	38.6	1.17e^{-3}
succinyl-diaminopimelate desuccinylase	DN14376_c0_g1_i1	12.7	0.03
sulfonate ABC transporter permease	DN15690_c0_g1_i1	10.2	0.03
TerC family protein	DN11390_c0_g1_i1	10.8	0.05
tetratricopeptide repeat protein	DN14826_c0_g1_i2	16.4	8.26e^{-3}
thioredoxin-dependent thiol peroxidase	DN10526_c0_g1_i1	10.8	0.05

	threonine--tRNA ligase	DN10111_c0_g1_i1	11.8	0.04
	thymidylate kinase	DN14879_c0_g1_i1	13.2	0.03
	TIGR02300 family protein	DN20462_c0_g8_i1	13.9	0.03
	translation initiation factor IF-3	DN14949_c0_g2_i1	14.6	0.03
	trigger factor	DN13945_c0_g1_i1	11.5	0.04
	tRNA(5-methylaminomethyl-2-thiouridylate)- methyltransferase	DN53941_c0_g1_i1	17.1	0.05
	tRNA-dihydrouridine synthase A	DN14326_c0_g1_i1	14.2	0.02
	two-component system sensor histidine kinase AtoS	DN14305_c0_g2_i1	20.4	0.04
	type I methionyl aminopeptidase	DN13945_c0_g2_i1	16.4	$7.51e^{-3}$
	ubiquinone biosynthesis protein UbiB	DN13521_c0_g1_i1	9.8	0.03
****A2*CLas*+ x A3*CLas*+**	3-oxoacyl-ACP synthase	DN17762_c4_g2_i2	36.8	$1.85e^{-3}$
	bordetella phage Bbp38 like protein	DN15458_c0_g2_i1	9.6	0.03
	cell wall-associated hydrolase	DN19816_c1_g6_i1	42.2	0.02
	Chloramphenicol-sensitive protein rarD	DN13473_c0_g1_i1	7.5	0.05
	DEAD/DEAH box helicase	DN4052_c0_g1_i1	9.7	0.02
	dicarboxylate/amino acid:cation symporter	DN14605_c0_g1_i1	7.0	0.04
	DNA topoisomerase IV subunit B	DN10717_c0_g1_i1	8.2	0.05
	ferrochelatase	DN15257_c0_g1_i1	7.0	0.01
	glycerol kinase	DN16085_c2_g1_i12	38.8	$2.50e^{-4}$
	glycerol kinase	DN16960_c2_g1_i14	24.0	$3.44e^{-3}$
	glycine/betaine ABC transporter substrate-binding protein	DN15178_c0_g1_i1	135.0	$3.57e^{-4}$
	helix-turn-helix transcriptional regulator	DN15227_c0_g1_i1	36.9	0.05
	HsdR family type I site-specific deoxyribonuclease	DN14127_c0_g1_i1	11.3	$5.14e^{-3}$
	hypothetical protein	DN10205_c0_g1_i1	13.1	0.02
	hypothetical protein	DN14146_c0_g1_i1	7.3	0.06
	hypothetical protein	DN17456_c0_g1_i4	7.1	0.02
	phage repressor protein/Prophage antirepressor	DN15458_c0_g2_i5	16.5	$5.14e^{-3}$
	phenylalanine--tRNA ligase subunit beta	DN14949_c0_g1_i2	19.9	0.02
	SAM-dependent methyltransferase	DN13350_c0_g1_i2	38.0	$2.35e^{-4}$
	threonine--tRNA ligase	DN10111_c0_g1_i1	8.9	0.01

*A1*CLas*$^{+}$: adults that fed on CLas-infected citrus plant for 1-2 days;
**A2*CLas*$^{+}$: adults that fed on CLas-infected citrus plant for 3-4 days; and
***A3*CLas*$^{+}$: adults that fed on CLas-infected citrus plant for 5-6 days.
